# Biology and Therapeutic Potential of Exosomes, Targeted Drug Delivery

**DOI:** 10.3390/ijms27188145

**Published:** 2026-09-12

**Authors:** Francisco Antonio Guillermo Mateos-Ramírez, Luis Daniel Hernández-Ortega, Carmen Magdalena Gurrola-Díaz, Luz Elena Gasca-Lozano, Laura Verónica Sánchez-Orozco, Adriana María Salazar-Montes

**Affiliations:** 1 Instituto de Investigación en Enfermedades Crónico-Degenerativas, Centro de Universitario de Ciencias de la Salud, Universidad de Guadalajara, Sierra Mojada 950, Guadalajara 44340, Jalisco, Mexico; francisco.mateos6114@alumnos.udg.mx (F.A.G.M.-R.); carmen.gurrola@academicos.udg.mx (C.M.G.-D.); luz.gasca@academicos.udg.mx (L.E.G.-L.); laura.sorozco@academicos.udg.mx (L.V.S.-O.); 2 Centro de Investigación Multidisciplinario en Salud, Centro Universitario de Tonalá, Universidad de Guadalajara, Av. Nuevo Periférico 555, Tonalá 45425, Jalisco, Mexico; luis.hortega@academicos.udg.mx

**Keywords:** exosomes, small extracellular vesicles, intercellular communication, targeted drug delivery, exosomes loaded, regenerative medicine, targeted therapy, therapeutic applications

## Abstract

Exosomes are small vesicles released by various cell types—compounds of a lipid bilayer and surface proteins that facilitate their recognition, direction, and uptake by the target cell. Inside, exosomes contain proteins, lipids, and nucleic acids, the composition of which varies depending on the cell of origin. Exosomes can be internalized by the target cell by endocytosis, fusion, or ligand–receptor interaction. The main functions of these extracellular vesicles (EVs) are intercellular communication, gene regulation, and the activation of cell regeneration and repair processes. Due to these characteristics, exosomes emerge as a promising option for the treatment of various diseases. Therefore, this review addresses the biology of exosomes and provides examples of studies with the molecular mechanisms by which these vesicles are taken up by target cells; examples of factors that influence the secretion and molecular composition of exosomes, which can be modified through exosome engineering, to enhance their effects. Furthermore, it addresses a diverse range of examples of the potential therapeutic applications of exosomes, including those loaded with different types of molecules, to use them as vehicles for drug delivery, with the objective of achieving safer, more precise, and effective treatments. Finally, it includes some clinical studies conducted with exosomes.

## 1. Introduction

Exosomes are a type of extracellular vesicle (EVs) secreted by various cell types, particularly mesenchymal stem cells (MSCs). Once released, EVs are detectable in a wide range of body fluids, including amniotic fluid, saliva, breast milk, blood, plasma, urine, cerebrospinal fluid, and semen. These vesicles typically range from 30 to 150 nm in diameter. Structurally, EVs feature a lipid bilayer membrane with integral and surface proteins, while their interior contains an array of bioactive molecules such as proteins, lipids, and nucleic acids, including DNA, mRNA, microRNAs, and both small and large non-coding RNAs [1,2,3,4,5]. The biogenesis of EVs occurs through distinct formation mechanisms depending on their biogenesis, such as exosomes, ectosomes (or microvesicles [MVs]), and apoptotic bodies [6,7]. Exosomes are derived from endosomal multivesicular bodies, which fuse with the plasma membrane [8]. Ectosomes are generated by budding outward from the plasma membrane, whereas apoptotic bodies appear only when cells are in the late stage of apoptosis [7].

However, in accordance with the Minimal Information for Studies of Extracellular Vesicles (MISEV) guidelines, it is critical to note that most widely used isolation techniques recover a heterogeneous mixture of vesicles rather than purely endosome-derived entities. Consequently, many literature reports traditionally referred to as “exosome studies” actually utilize heterogeneous Small Extracellular Vesicles (sEVs) preparations, unless specific endosomal biogenesis pathways are experimentally demonstrated. Accordingly, the International Society for Extracellular Vesicles (ISEV) recommends using the generic term “EV” and the operational terms “Large extracellular vesicles” (LEVs) and “small extracellular vesicles” (sEVs), with the latter including small ectosomes and exosomes [9].

However, it is crucial to emphasize that these groups do not represent entirely distinct or isolated populations; instead, they form a highly heterogeneous biological continuum. Because of significant overlap in size distributions, physical morphology, and biochemical markers, none of these criteria alone are sufficient to classify or identify a specific vesicle subtype. Consequently, current guidelines recommend utilizing operational terms like “small EVs” unless specific biogenesis pathways are experimentally demonstrated. (Table 1). It is worth noting that the research considered in this review—referred to as “exosome studies”—may have utilized heterogeneous preparations of small extracellular vesicles.

EVs facilitate contact-independent intercellular communication. Signaling initiates when donor cells release sEVs into the extracellular space, enabling their migration through body fluids to target cells or injured tissues. They facilitate paracrine signaling by transporting bioactive molecules, thereby influencing cellular function [10,11,12]. The sEVs composition and, therefore, activity, are dictated by their cellular origin. It is worth noting that, depending on their origin, they may exhibit very low immunogenicity [12]. Furthermore, their involvement in embryogenesis, immune modulation, lactation, and neuronal regulation is well-established. Their contribution has also been investigated in disorders such as neurodegeneration and malignancy [10]. Due to the pivotal role that EVs play at the cellular level, they are being studied as therapeutic agents for multiple diseases [11]. Currently, there is great interest in using them as vehicles for drug delivery in targeted therapy applications, with the principal aim of achieving safer, more precise, and effective treatments [3,13,14,15].

## 2. Exosome Biogenesis

Exosomes biogenesis begins with endocytosis, initiated by cell membrane invagination via either clathrin-mediated or clathrin-independent mechanisms [2,5,12]. The resulting endocytic vesicle detaches and is transported intracellularly to form an early endosome, which subsequently matures into a late endosome [16]. During this maturation, the endosome develops into a multivesicular body (MVB), a process characterized by the inward budding of its membrane to form intraluminal vesicles (ILVs). Throughout both early and late stages, endosomes undergo cargo sorting; consequently, their structure and composition depend strictly on the donor cell and microenvironmental conditions [5,16]. Ultimately, MVBs follow one of two pathways: they either fuse with lysosomes to degrade their internal cargo, or they are transported to and fuse with the plasma membrane, releasing the ILVs into the extracellular environment as exosomes [5,16].

### 2.1. Endosomal Sorting Complex Required for Transport Machinery

**Figure 1:**
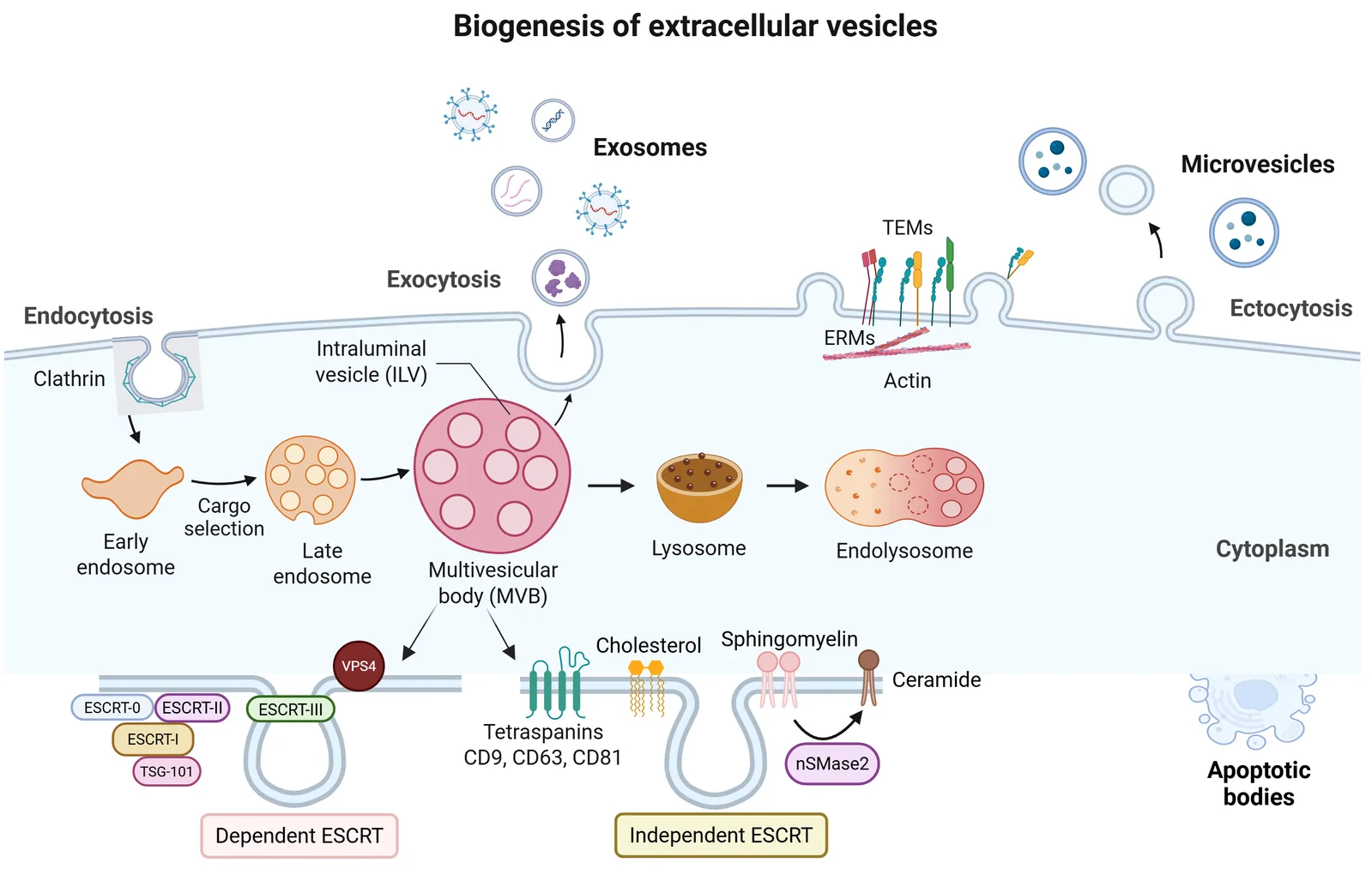
EVs biogenesis. Formation of ILVs, ESCRT-dependent pathway and ESCRT-independent pathway. Tetraspanin-enriched microdomains (TEMs); Ezrine, Radixine, Moesine (ERMs); Neutral sphingomyelinase 2 (nSMase2). “Created in BioRender. Mateos, F. (2026) https://BioRender.com/hvjtudx”.

The endosomal sorting complex required for transport (ESCRT) is fundamental to the regulation of exosome biogenesis. It consists of four distinct complexes (ESCRT-0, -I, -II, and -III) along with associated accessory proteins (VPS4, VTA1, and ALIX) that collectively participate in recognizing exosome-targeting proteins and driving MVB formation [17,18]. The ESCRT-0 complex comprises two primary components: the hepatocyte growth factor-regulated tyrosine kinase substrate (Hrs) and the signal transducer adaptor molecule (STAM, specifically the STAM1 and STAM2 isoforms). Both subunits bind to ubiquitin, providing an additional targeting module that promotes their recruitment to cargo-enriched endosomes. Recruitment of ESCRT-I by ESCRT-0 is essential for initiating cargo sorting during MVB formation [17]. The ESCRT-I complex (featuring TSG101) interacts with both ESCRT-0 and ESCRT-II at opposite ends of the complex. ESCRT-II is composed of Vps36, Vps22, and Vps25. Together, ESCRT-I and -II initiate the budding process and promote the enzymatic deubiquitination of cargo proteins before the formation of ILVs, which aggregate within the intracellular compartment to form larger membranous vesicles called MVBs [19,20]. Finally, the ESCRT-III complex—composed of Vps20, multivesicular body protein 4 (CHMP4), Vps24, and Vps2—participates in membrane invagination and subsequent cleavage. Concurrently, Vps4 exerts AAA-ATPase activity to hydrolyze ATP, leading to the disassembly and recycling of ESCRT-III components; it also plays a critical role in ILV loading and cargo sorting [19,20] (Table 2, Figure 1).

The Rab protein family is composed of small GTPases belonging to the RAS superfamily. Their primary function is to coordinate MVB transport, docking and fusion with the plasma membrane. Each member of the Rab GTPases family exhibits a specific subcellular location, and their activation enables participation in membrane trafficking, vesicle formation, motility, and fusion. During early endosome maturation, transmembrane proteins are recycled to the plasma membrane via Rab2B and Rab4, whereas Rab5 mediates endocytosis alongside the generation and maintenance of early endosomes [21].

Rab27 is formed by the Rab27A and Rab27B subunits. Rab27A primarily mediates the docking, tethering, and fusion of MVBs with the plasma membrane, and silencing of these genes has been directly associated with the inhibition of exosomes secretion. Additionally, Rab11 handles the transport of recycling endosomes, promoting their formation and subsequent exocytosis. Meanwhile, Rab35 facilitates docking and regulates the anchoring of MVBs to the plasma membrane. Finally, Rab7 is responsible for coordinating trafficking between late endosomes and lysosomes. The final step for the release of MVBs into the extracellular space and the formation of exosomes is the fusion of the MVB membrane with the cell membrane. Studies have shown that this process depends on the SNARE protein family. SNARE proteins include (vesicular SNARE, v-SNARE), and (target SNARE, t-SNARE). These proteins mediate the specific recognition and fusion of the transport vesicle membrane with the target membrane [21].

### 2.2. Transport-Independent Endosomal Sorting Complex

Depletion of subunits belonging to the four ESCRT complexes does not completely abolish MVB formation, indicating that alternative mechanisms may be involved in ILVs biogenesis. One such alternative route is the ceramide/tetraspanin pathway [2,12,19]. A previous study demonstrated that the introduction of synthetic ceramide can facilitate MVBs budding through its metabolism into sphingosine-1-phosphate (SIP), which subsequently binds to the S1P receptor on MVBs to stimulate ILV production [22]. Additionally, tetraspanin proteins (CD81, CD82, and CD9), which are highly enriched in exosomes, are intimately involved in exosomes sorting and loading. These proteins interact with various receptors within the plasma membrane, driving the formation of specialized microdomains [12] (Figure 1).

**Table 2:** Endosomal sorting complex required for transport (ESCRT) proteins.

Complexes	Subunits	Function	References
ESCRT-0	HRS, STAM1/2	Recognition and recruitment of proteins for internalization (ubiquitinated proteins, clathrin) and TSG101.	[19,20]
ESCRT-I	TSG101, VPS28, VPS37, MVB12	Initiation of budding and promotion of cargo deubiquitination prior to ILV formation within MVBs; recruitment of ESCRT-III by ESCRT-I via ESCRT-II or ALIX.	[19,20]
ESCRT-II	VPS36, VPS22, VPS25		
ESCRT-III	VPS2, VPS4, VPS20, CHMP4	Invaginate the membrane and drive subsequent vesicle scission.	[19,20]
AAA ATPases.	VPS4	Hydrolyzes ATP to drive disassembly and recycle ESCRT-III components. Interacts with ESCRT-III to promote constriction and cleavage of the ILVs.	[23]
ESCRT-associated proteins.	ALIX	Cargo control, regulation, and PD-L1 sorting into ILVs; stimulation of intraluminal budding by ALIX and the syntenin–ALIX complex.	[24]

### 2.3. Molecular Mechanisms of Cargo Sorting

The enrichment of specific proteins, RNAs, and lipids within exosomes is not a random process but is tightly regulated by distinct, interconnected molecular sorting machinery. Understanding these pathways is paramount for the design and optimization of therapeutic exosomes.

#### 2.3.1. RNA Cargo Sorting: Motifs and RNA-Binding Proteins (RBPs)

The selective compartmentalization of miRNAs into exosomes is largely dictated by specific nucleotide sequence patterns, known as EXOmotifs (e.g., GGAG or CCCU), present within the RNA secondary structure. These motifs are recognized by specialized RNA-binding proteins (RBPs) that facilitate their transport into nascent vesicles:

hnRNPA2B1 (Heterogeneous Nuclear Ribonucleoprotein A2B1): This RBP specifically binds to the GGAG motif on target miRNAs. Crucially, this interaction is regulated by sumoylation; the sumoylated form of hnRNPA2B1 directs the bound miRNA into the invaginating endosomal membrane. For its part, sumoylated hnRNPA1 recognizes the GAGAG motif in the 3′ region of the miRNA and loads these molecules into EVs. The Argonaute 2 protein (AGO2) is a component of the miRISC complex that regulates miRNA formation, and degradation also participates in miRNA selection for EVs. It was demonstrated that AGO2 gene deletion (knockout) reduced the levels of several miRNAs, such as miR-451, miR-150, and miR-142-3p, in EVs. Regarding YBX1 (Y-Box Binding Protein 1) acts as another vital chaperone, particularly sorting non-coding RNAs and specific miRNAs such as miR-223 into exosomes through liquid–liquid phase separation and direct binding dynamics [25,26].

#### 2.3.2. Lipid-Mediated Sorting

Exosome sorting and formation do not rely solely on the ESCRT system but involve lipid- and protein-mediated mechanisms that reflect the heterogeneity of these vesicles. Lipid rafts act as platforms for selectively sorting, clustering, and loading proteins and lipids into ILVs. Ceramide, a sphingolipid, is crucial for loading specific proteins (such as proteolipid protein) and triggers membrane invagination to form the vesicles. Meanwhile, tetraspanin proteins like CD63 and CD9, along with their associated lipid-enriched domains, facilitate the organization and loading of transmembrane proteins into exosomes [27].

#### 2.3.3. Protein Sorting

Post-translational modifications are key regulators of the mechanisms governing protein sorting and selective enrichment in exosomes. While processes such as ubiquitination and sumoylation promote the incorporation of proteins into the exosomal cargo, modifications like acetylation and isgylation act in the opposite manner, redirecting them toward degradation pathways. Likewise, phosphorylation and glycosylation alter the biogenesis and molecular composition of both the cargo and the exosomal membrane [27].

## 3. Molecular Composition of Exosomes

**Figure 2:**
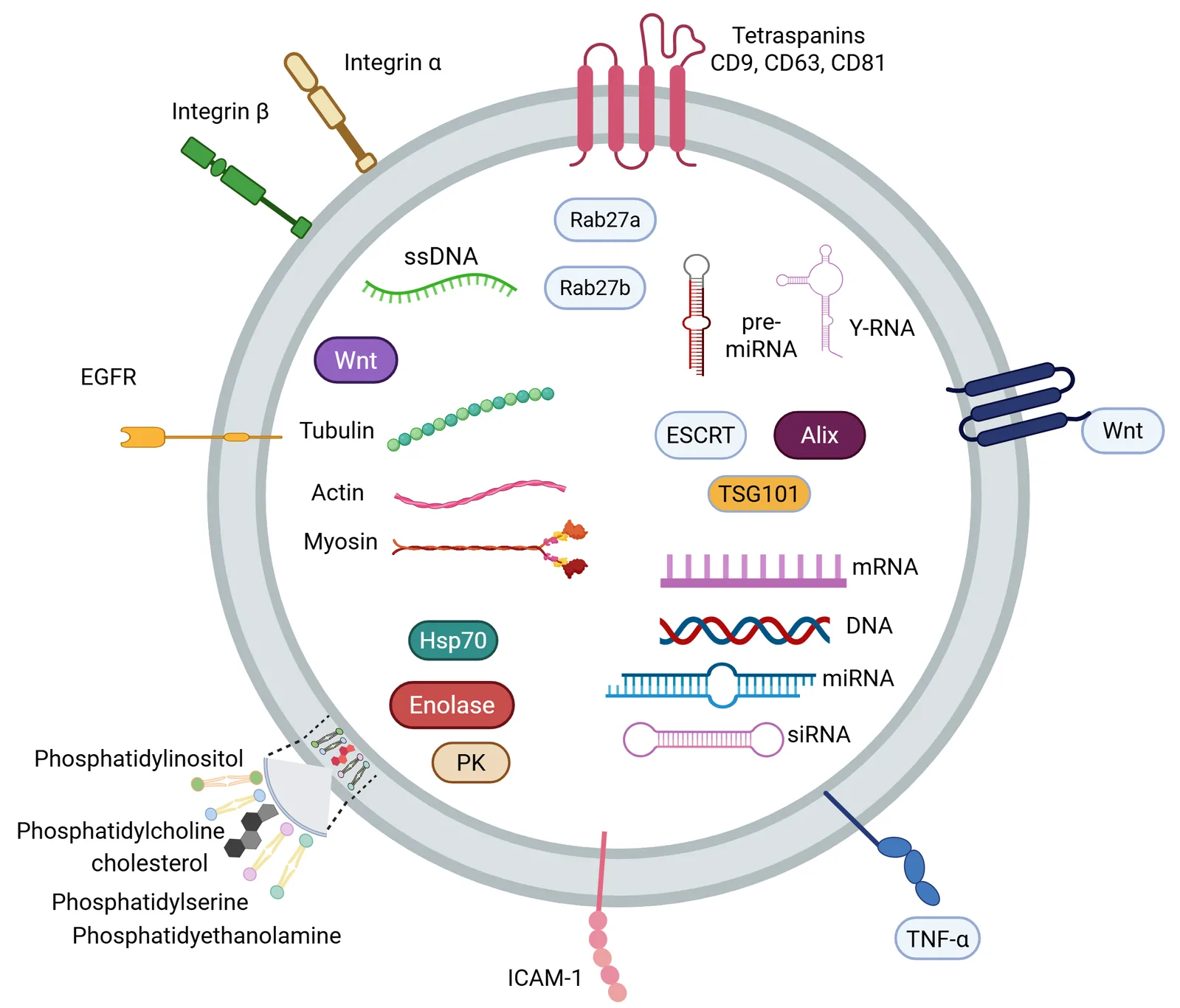
Structure and composition of exosomes. “Created in BioRender. Mateos, F. (2026) https://BioRender.com/tjmgr57”.

Exosomes contain approximately 194 lipids, 8000 proteins [6], 764 miRNAs, and 1639 mRNA types, depending on the cell of origin [28,29] (Figure 2). The composition of exosomes can be affected by factors such as the microenvironment, topography, cell of origin [30], medium composition, mechanical stress, disease status, and cellular oxygen levels in the cell [31,32].

Some proteins on exosomes can be used as markers to characterize them [33]. Examples of these markers include tetraspanin, CD81, CD63, and CD9, which participate in protein trafficking and membrane fusion, as well as TSG101, flotillin, and ALIX, which engage in their biogenesis [1,5,12]. Although these proteins are commonly referred to as “classic exosome markers,” they are not strictly exclusive to endosome-derived exosomes, as they are also associated with EVs in general.

### 3.1. Lipids

Exosome membranes are composed of several types of lipids, including cholesterol, phosphatidylserine (PS), sphingomyelin [34], phosphatidylcholine (PC), phosphatidylethanolamine (PE), phosphatidylinositol (PI), phosphatidic acid (PA), ceramides, glycosphingolipids, and other less abundant lipids [35]. The lipid concentration in the exosome membrane differs from that of plasma and cell membranes due to exosomes biogenesis [1]. These lipids determine the fate and bioactivity of the vesicles. Through them, exosomes can interact with proximal or distal cells and exert effects on target cells. Lipid receptors on target cells can also determine the fate of exosomes. For example, PS can be recognized by target cells through their TIM cell surface receptors (T-cell immunoglobulin and mucin receptors). Lysophosphatidylcholine (LPC) can be recognized by the G protein-coupled receptor G2A. PS can bind to both TIM-1 and TIM-4 receptors and regulate certain immunological processes and the elimination of apoptotic cells. Furthermore, LPC can attract T lymphocytes via chemotaxis and participate in dendritic cell maturation and the immune response [11].

ILVs, precursors of exosomes, evolve into exosomes through microautophagy with the participation of ESCRT, the heat shock protein Hsc-70, and the sorting protein Vps4. All of these interact with lipid metabolism and participate in exosome production [36]. In this regard, cholesterol, oxysterols, ceramides, and lipid transporters have been shown to participate actively in the exosome production process [11].

### 3.2. Proteins

Membrane proteins of MSC-derived exosomes include glycosylphosphatidylinositol-anchored proteins, such as CD73, and tetraspanins such as CD9, CD37, CD53, CD63, CD82, and CD81. Other proteins involved in exosome transport and fusion to the target cell membrane are annexins I, II, IV, V, and VI, and Rab GTPases, as well as antigen-presenting proteins such as MHC-I, MHC-II, and CD86. Cell adhesion molecules, such as integrins, are also present, along with structural and motility proteins like actin, myosin, and tubulin. Inside exosomes, we find heat shock proteins such as HSC70, HSP20, HSP60, HSP70, HSP84, and HSP90, chaperones such as the β subunit of the TCP-1 chaperone family, the γ subunit of the TCP-1 chaperone family, and the ε subunit of the TCP-1 chaperone family [1,5,11,12]. Some metabolic enzymes, such as pyruvate kinase, enolase 1, fatty acid synthase, peroxidases, glyceraldehyde-3-phosphate dehydrogenase, phosphoglycerate kinase 1, citrate synthase, malate dehydrogenase, and fatty acid-binding proteins 3 and 4, have also been reported alongside proteins related to exosome biogenesis, such as the ESCRT complex, ALIX, and TSG101. Other documented components include proteins involved in transcription and translation, such as, Translation Elongation Factor 1 and 2, and Translation Initiation Factor 4A; ubiquitin/proteasome-related proteins, nuclear proteins such as histones (histone H1.1, H1.3, H1.5, H2A, H2B and H4), ribosomal proteins (ribosomal Protein 40S SA, 40S S8, 60S L7, and 60S L18a) and transcription factors [1,5,11,12]. Exosomes also contain surface proteins involved in cell signaling. These include wingless proteins (Wnt), crucial in embryonic development and adult tissue homeostasis, and their receptor GPR177, which regulates cell proliferation and differentiation [37]. In addition, exosomes have been described as carriers of bone morphogenetic proteins bound to the EV surface [7], transforming growth factor β1 (TGF-β1) [38], tumor necrosis factor α (TNF-α) [39], the first signal of apoptosis ligand (FAS) [40], cytokines [41], and other signaling molecules, reinforcing the idea that exosomes are messengers capable of transmitting complex autocrine and paracrine signals. The exosomes surface is also rich in extracellular matrix (ECM) proteins, such as fibronectin and tenascin C, which also play important roles in cell signaling and adhesion [42,43,44].

### 3.3. RNA

RNA represents a considerable fraction of the exosomes content. Among the various subtypes, exosomes contain microRNA (miRNA), messenger RNA (mRNA), transfer RNA (tRNA), ribosomal RNA (rRNA), small interfering RNA (siRNA), small nuclear RNA (snRNA), Y-RNA, and vault RNA (vtRNA) [45], all of which possess the capacity to induce biological effects. The exosomal RNA profile varies dynamically depending on the cell type of origin; for instance, MSC-derived exosomes typically carry RNAs related to immunomodulation, cell survival, and differentiation [12]. In particular, mRNA within bone marrow mesenchymal stem cell-derived exosomes (BM-MSCs-Ex) are associated with mesenchymal phenotype differentiation, transcription, and cell proliferation [46], whereas those EVs derived from human liver stem cells are linked to metabolic and proliferative pathways [47]. Consequently, a large number of studies on exosome-mediated cell communication focus primarily on miRNAs and mRNAs [45].

miRNAs are small non-coding RNA molecules that regulate gene expression in recipient cells. Their sorting into exosomes depends on the stimuli received by the parent cell and the surrounding microenvironment; therefore, exosomal miRNA content reflects the specific stress or disease conditions experienced by the individual [11]. In contrast, siRNAs participate in the inhibition of gene expression and thus play a key role in gene silencing [48]. Y-RNAs bind to Ro protein to regulate RNA stability and participate in the initiation of DNA replication [49,50]. Vault RNAs (vtRNAs) are highly conserved in eukaryotes; however, a firm consensus on their exact function has yet to be established. Accumulated evidence suggests that vtRNAs can act similarly to miRNAs by binding to Argonaute proteins and inhibiting target gene expression [51].

### 3.4. DNA

Exosomes contain various forms of DNA, including single-stranded, double-stranded, genomic, and mitochondrial DNA. This mitochondrial DNA can be complete or fragmented and can be transferred to cells with altered metabolism to restore their metabolic activity. For instance, a study on breast cancer cells demonstrated that hormone therapy induced a deficiency in oxidative phosphorylation, which was subsequently restored via the transfer of mtDNA-loaded exosomes, leading to increased self-renewal and hormone therapy resistance [52,53,54]. Furthermore, exosomal double-stranded DNA, which spans the entire genome, can be used to identify mutations present in parental tumor cells. This highlights its potential utility as a circulating biomarker for cancer diagnosis in clinical practice [55] (Figure 2).

## 4. Cellular Sources of Exosomes

Exosomes can be isolated from various cellular and tissue sources, including menstrual blood, dental pulp, skin, the umbilical cord, adipose tissue, placenta, bone marrow, and immune cells [6,56,57]. Among these, mesenchymal stem cells (MSCs), which are multipotent, non-hematopoietic cells residing in adult tissues that regulate tissue homeostasis, repair, and regeneration represents one of the most prominent sources [58,59,60]. Due to their immunomodulatory properties, MSCs are widely utilized as cell therapies for various diseases [61]. These cells can differentiate in multiple lineages, such as osteocytes, endothelial cells, and chondrocytes, with ample evidence of their therapeutic potential [62]. The secretome released by the cells into the extracellular environment includes exosomes, EVs, cytokines, and growth factors [63]. MSC-derived exosomes actively participate in intercellular communication, which permits us to understand their functional roles under physiological and pathological conditions [64]. Under homeostatic conditions, these EVs exert anti-apoptotic, anti-inflammatory, and antioxidant effects [65].

### 4.1. Exosomes Derived from Bone Marrow Mesenchymal Stem Cells

Bone marrow-derived mesenchymal stem cells (BM-MSCs) share multilineage differentiation potential with other MSCs, readily committing to various mesodermal cell types. Isolating BM-MSCs requires a bone marrow aspirate, typically harvested from the iliac crest or the metaphysis of the distal femur and proximal tibia. Exosomes secreted by BM-MSCs (BM-MSC-Ex) exhibit a characteristic discoid morphology with a diameter ranging from 30 to 100 nm. Characteristically, they express classical endosomal markers such as CD63, CD9, and TSG101, alongside enriched levels or miR-96 [66,67].

### 4.2. Exosomes Derived from Adipose Tissue Mesenchymal Stem Cells

Adipose tissue-derived mesenchymal stem cells (AMSCs) can differentiate into adipocytes, osteoblasts, chondrocytes, and myocytes, making them highly valuable for tissue engineering applications, such as bone fracture healing [68,69]. AMSCs are typically harvested from adipose tissue via liposuction. Exosomes derived from these cells (AMSC-Ex) range from 30 to 200 nm in size and are cup-shaped under transmission electron microscopy (TEM). The presence of exosomal markers CD9, CD63, and CD81 has been identified on their surface [70,71].

### 4.3. Exosomes Derived from Umbilical Cord Mesenchymal Stem Cells

Like BM-MSCs, umbilical cord mesenchymal stem cells (UC-MSCs) possess high self-renewal capacity and low immunogenicity. However, they are obtained through completely non-invasive procedures and expand easily in culture, making them potentially superior for cell transplantation to other MSCs from other sources [72]. UC-MSC-derived exosomes (UC-MSC-Ex) have demonstrated immense promise in regenerative medicine. Under TEM these vesicles present a spherical morphology with a size distribution of 50–150 nm, and they consistently test positive for the membrane markers CD9, CD63, and CD81 [72,73,74,75,76].

## 5. Factors Involved in Exosomes Secretion

**Figure 3:**
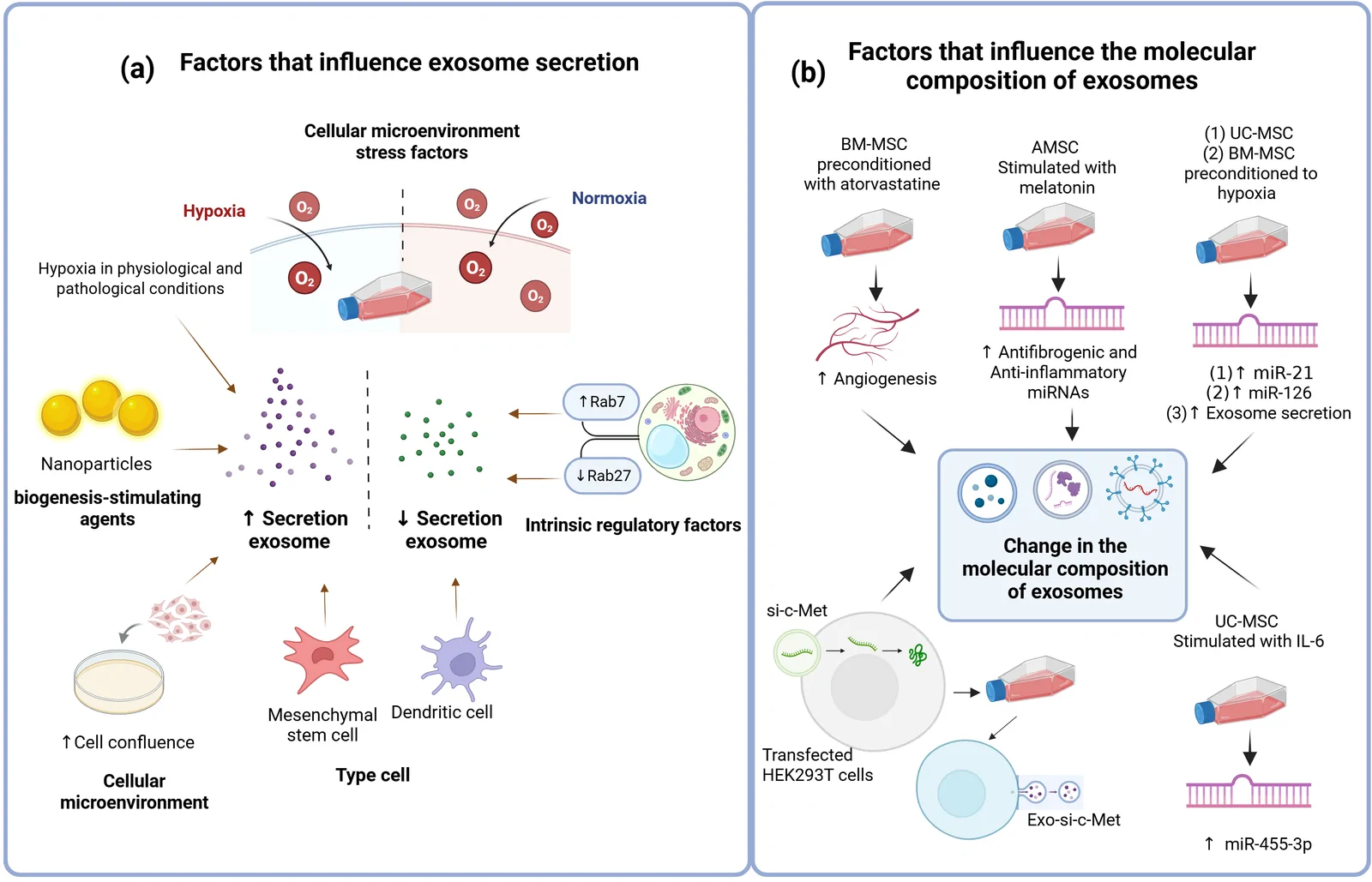
Factors involved in exosome secretion and molecular composition. (**a**) Factors that influence exosome secretion. (**b**) Factors that influence the molecular composition of exosomes. “Created in BioRender. Mateos, F. (2026) https://BioRender.com/ys4fh8k”.

Exosomes biogenesis and release are highly influenced by cell type, the microenvironment, and cell confluence [31]. Regarding cell type-specific dynamics, immature dendritic cells produce limited quantities of exosomes [77,78], whereas mesenchymal stem cells can secrete them abundantly, a characteristic that highlights their potential for therapeutic applications [79,80]. Mechanistically, exosome release depends on the expression of Rab27 and Ral proteins, which regulate vesicle trafficking and docking. Consequently, the fusion of multivesicular bodies (MVBs) with the plasma membrane and subsequent exosome secretion are significantly impaired in cells with depleted levels of these regulatory proteins [81]. Furthermore, exosomes secretion is modulated by extrinsic elements such as culture conditions, cytokines, growth factors, nanoparticles, and chemical stimuli [31]. For instance, supplementing MSC cultures with a combination of N-methyldopamine and noradrenaline markedly enhanced exosome production and secretion [82]. Furthermore, exosome release is influenced by calcium. A study conducted in K562 hematopoietic cells demonstrated this calcium-dependent process, regulated by both chemical and physiological stimuli. Key findings included the effect of monensin (MON), a Na^+^/H^+^ exchanger, which significantly increased exosome secretion and generated large MVBs with calcium accumulation. Increased intracellular calcium stimulated exosome secretion, while the presence of the intracellular chelating agent 1,2-bis(2-aminophenoxy) ethane-*N*,*N*,*N*′,*N*′-tetraacetic acid acetoxymethyl ester (BAPTA-AM) completely blocked MON-induced release. Physiologically, transferrin stimulated calcium-dependent exosome release in K562 cells [83]. Similarly, exposure to various nanomaterials, including platinum nanoparticles, has been shown to upregulate both exosome biogenesis and release [84] (Figure 3a).

The composition and secretion of sEVs are synchronized and do not function independently. The biological activity of secreted sEVs is determined by the specific molecular signature established prior to their extracellular release. Selective sorting mechanisms enrich these vesicles with specific proteins, lipids and nucleic acids. Consequently, the sEVs act as a direct molecular signal of the parent cell, carrying functional molecules optimized for targeted cell-to-cell signaling.

## 6. Factors Involved in the Molecular Composition of Exosomes

The release of exosomes is influenced not only by microenvironmental cues, including mechanical properties, topography, and biochemical stimuli [30]. For instance, cells cultured under hypoxic conditions exhibit increased exosome secretion, a phenomenon observed under both physiological and malignant conditions [32] (Figure 3a). In a preclinical study, the administration of exosomes derived from hypoxia-preconditioned BM-MSCs to APP/PS1 transgenic mice led to an upregulation of miR-21 expression. This molecular change correlated with a significant improvement in cognitive tests, alongside a marked reduction in plaque beta-amyloid (AB) plaque deposition, levels of soluble AB, and pro-inflammatory cytokines. Furthermore, the activation of the STAT3 and NF-κB signaling pathways was significantly down-regulated. The proposed mechanism suggests that the enrichment of miR-21 restored synaptic function and effectively modulated the inflammatory response [85].

Similarly, using both in vivo and in vitro bone fracture models, the administration of UC-MSC-Ex preconditioned under hypoxia promoted angiogenesis, cell proliferation, and migration to a greater extent than UC-MSC-Ex obtained under normoxic conditions. These hypoxic exosomes exhibited an upregulation of miR-126, a response likely driven by hypoxia-inducible factor 1-alpha (HIF-1α) [86].

Pharmacological preconditioning has also shown promising results. When diabetic rats with cutaneous wounds were treated with BM-MSC-Ex derived from cells pre-stimulated with atorvastatin, wound regeneration was accelerated via enhanced angiogenesis. Correspondingly, in vitro assays with endothelial cells demonstrated that these specific exosomes induced cell proliferation and triggered the secretion of vascular endothelial growth factor (VEGF). These pro-angiogenic effects were mediated by the activation of the AKT/eNOS signaling pathway [87].

In a murine model of chronic kidney disease, therapeutic intervention using melatonin-stimulated AMSC-derived exosomes led to a significant shift in the microRNA profile: microRNAs associated with anti-inflammatory and anti-fibrotic processes were significantly upregulated, whereas those driving disease progression were suppressed [88].

In addition to physiological and pharmacological stimuli, cytokine preconditioning can modulate exosomal cargo. For example, stimulating UC-MSCs with IL-6 induces the expression of miR-455-3p, an microRNA that regulates PI3K activity. Exosomes isolated from these cells and enriched with miR-455-3p effectively inhibited the activation of lipopolysaccharide (LPS)-stimulated macrophages and suppressed cytokine expression both in vitro and in vivo, ultimately mitigating acute liver injury in a murine model [89].

Finally, exosomes serve as highly efficient vehicles for targeted genetic delivery. In one study, BM-MSC-derived exosomes were engineered to deliver miR-146, a microRNA with well-documented antitumor properties, via plasmid transfection of the parental cells. Intratumoral administration of these miR-146-enriched exosomes significantly suppressed the growth of glioma xenografts in a murine primary brain tumor model [90]. Utilizing a similar approach, HEK293T cells were transfected with small interfering RNA against c-Met (si-c-Met). The resulting exosomes containing the therapeutic cargo (exo-si-c-Met) successfully inhibited invasion and migration while promoting apoptosis in gastric cancer cell lines in vitro and successfully suppressed tumor growth in vivo within a xenograft model [91] (Figure 3b).

## 7. Preparation and Characterization of Extracellular Vesicles

### 7.1. Cell Culture Conditions and Medium Composition

Cell culture protocols for exosome isolation vary considerably among laboratories, even when utilizing well-established cell lines. Each parental cell type requires a tailored medium composition to ensure optimal growth, alongside strict parameters for seeding density, passage frequency, and medium replenishment intervals dictated by maintenance requirements [92].

Crucially, both the passage number and the initial seeding density profoundly impact on the cargo composition and biological functions of secreted exosomes. Components within the culture medium further modulate exosome yield and molecular profile [92,93]. For instance, glucose concentration plays a pivotal role in EVs biogenesis; high glucose levels enhance EV production and stimulate the secretion of larger vesicles, ultimately altering their molecular composition [92,94].

Another critical factor is the addition of fetal bovine serum (FBS), a protein-rich supplement that carries the significant drawback of containing endogenous EVs, which can confound downstream in vitro or in vivo analyses. While transitioning to serum-free media is a common strategy, complete serum starvation can induce severe metabolic stress, thereby altering cell behavior and the cargo of secreted EVs. To circumvent this issue, the utilization of commercially available, exosome-depleted FBS is highly recommended [92].

### 7.2. Isolation of Extracellular Vesicles

Ideally, the isolation process should yield high-purity exosomes with optimal recovery rates. Exosomes can be isolated from a diverse array of sources, including milk, cerebrospinal fluid, saliva, urine, serum, plasma, and cell culture media [95] among these, cell culture supernatants typically offer the highest yield [96,97]. Consequently, large-scale exosome production heavily relies on cell culture media, as this approach is straightforward, cost-effective, and circumvents the ethical and logistical constraints of utilizing animal or human subjects [92]. However, exosomes are inherently heterogeneous in size, cargo, function, and biogenesis [98]. The co-existence of confounding components with similar properties, such as cellular debris and protein aggregates within the medium or biological matrix, poses a major challenge to achieving high-purity isolation [3,99].

To address these challenges, researchers have developed various isolation methodologies. These include ultracentrifugation, size-based separation techniques (such as size-exclusion chromatography), ultrafiltration, polymer-based precipitation, and immunoaffinity capture techniques [3,6,13,100,101]. As previously mentioned, it is important to highlight that these isolation techniques typically yield mixed and heterogeneous populations of EVs.

**Figure 4:**
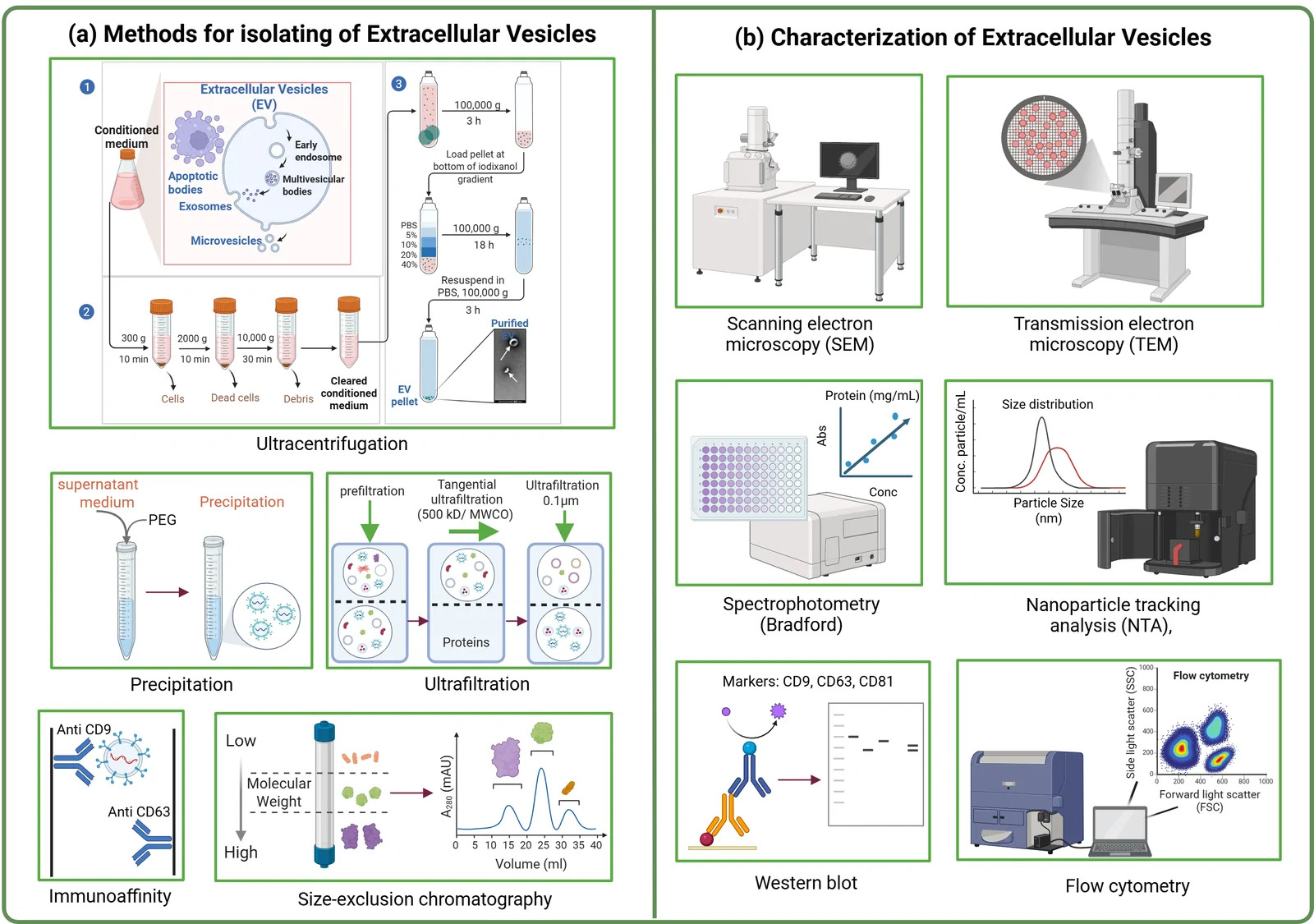
Preparation and characterization of EVs. (**a**) Methods for EVs isolation. (**b**) Techniques for EVs characterization. “Created in BioRender. Mateos, F. (2026) https://BioRender.com/ofg951i”.

As compiled in Table 3, selecting an isolation methodology requires a deliberate compromise between sample yield and final purity. While precipitation-based methods prioritize high particle recovery at the expense of purity, techniques such as Size Exclusion Chromatography (SEC) and immunoaffinity capture offer superior clean sEV preparations but face challenges regarding volume scalability and absolute yield. Understanding these specific limitations is paramount, as the choice of isolation technique directly influences the downstream molecular profile and therapeutic potency of the recovered vesicles (Table 3, Figure 4a).

### 7.3. Characterization and Identification of Extracellular Vesicles

Following isolation, validation of purified vesicles is mandatory to guarantee the presence, identity, quantity, and purity of the preparation. The use of protein indicators alone is insufficient to classify these populations. There is a strict necessity for orthogonal characterization, which entails coupling biochemical marker analysis with biophysical validation methodologies. Current characterization methodologies comprise a combination of biophysical, imaging, and molecular techniques. The parameter for characterization include flow cytometry and immunoaffinity-based capture (IAC) are frequently used to analyze surface protein profiles, and the most used Western blot to confirm the detection of positive indicators (such as transmembrane tetraspanins CD9, CD63, and CD81, or cytosolic proteins like Alix and TSG101) and negative indicators (such as calnexin, GM130, or cytochrome c), which rule out cellular debris or contamination from specific intracellular compartments. And enzyme-linked immunosorbent assay (ELISA) offers a highly specificity, rapid, and suitable for high-throughput screening of specific protein markers. Other techniques of imaging to evaluate size and morphology, such as atomic force microscopy (AFM), scanning electron microscopy (SEM), and transmission electron microscopy (TEM) are widely utilized, with TEM remaining the conventional gold standard. Specifically, SEM enables the direct observation of surface topography, whereas TEM provides high-resolution insights into both the internal structure and structural integrity of individual vesicles, alongside particle-tracking technologies (e.g., Nanoparticle Tracking Analysis [NTA] or Interferometric Light Microscopy) to determine size distribution and concentration. Finally, total protein in vesicle preparation quantification is routinely achieved using colorimetric assays, such as the Bradford and bicinchoninic acid (BCA) protein assays [3,4,6,9,99,101]. Only through this multi-layered, orthogonal approach can the purity and identity of EV preparations be reliably established (Figure 4b).

## 8. Exosome Uptake by the Target Cell

### 8.1. Mechanisms of Exosome Uptake

Once sEVs reach their target destinations, their therapeutic payload must be internalized by recipient cells to exert a biological effect. Emerging evidence demonstrates that sEVs uptake is an extraordinarily heterogeneous and dynamic process. A single population of sEVs typically utilizes multiple, parallel endocytic and non-endocytic pathways simultaneously, heavily dictated by the recipient cell type, its metabolic state, and the specific molecular topography of both vesicle and host membranes. Internalization pathways can be broadly categorized into three distinct, non-mutually exclusive mechanisms:

#### 8.1.1. Direct Plasma Membrane Fusion

EVs can directly fuse with the plasma membrane of the target cell, discharging their luminal cargo into the cytoplasm. Mechanistically, when the exosomal lipid bilayer comes into close apposition with the target cell membrane, it forms an intermediate hourglass-shaped structure termed the fusion stalk (or hemifusion diaphragm). As this stalk expands, the outer leaflets merge into a single structure (the hemi-bilayer). Subsequent expansion and tension within this hemifusion zone induce the rupture of the inner lamellae, opening a transient fusion pore that directly connects the exosomal lumen with the cytoplasm of the target cell [103,104]. This fusion-mediated entry facilitates the functional transfer of exosomal mRNA and miRNA, a phenomenon robustly validated by tracking RNA transport from mouse mast cell-derived exosomes into human recipient mast cells. Notably, this transfer led to the successful translation and detection of mouse-specific proteins within the human recipient cells, definitively demonstrating that exosomal mRNA remains stable and translationally active upon intracellular delivery [105].

#### 8.1.2. Endocytosis

Alternatively, EVs can enter recipient cells via endocytic pathways, which are broadly categorized into clathrin-dependent and clathrin-independent mechanisms.

##### Clathrin-Mediated Endocytosis (CME)

This process initiates when specific receptors on the target cell membrane recognize exosomal surface ligands or adhesion molecules. This interaction drives the assembly of clathrin-coated pits, a process orchestrated by adaptor proteins (such as adaptin) that recruit clathrin triskelions to the cytosolic face of the membrane. The pit then invaginates inward and is pinched off from the plasma membrane by the GTPase dynamin-2, releasing the vesicle into the cytoplasm [104]. CME-mediated uptake has been widely documented in epithelial cells, neurons, hepatocytes, cardiomyocytes, and macrophages, as well as in colorectal and ovarian cancer cells [106,107].

##### Clathrin-Independent Endocytosis

These alternate pathways bypass clathrin assembly and are subdivided into lipid raft-mediated endocytosis, caveolin-dependent endocytosis, phagocytosis, and macropinocytosis [2,103,104].

- Lipid Raft-Mediated Endocytosis: This pathway is characterized by highly dynamic microdomains within the plasma membrane that are enriched with sphingolipids, cholesterol, and glycosylphosphatidylinositol-anchored proteins. Consequently, disrupting lipid metabolism or depleting membrane cholesterol severely impairs exosome internalization through these specialized domains.
- Caveolin-Dependent Endocytosis: Caveolae represents a specialized subset of glycolipid rafts, presenting as small flask-shaped invaginations of the plasma membrane. Their formation depends structurally on caveolins (integral membrane proteins). These subdomains facilitate stable interactions with EVs, subsequently triggering their internalization.
- Phagocytosis: Dendritic cells and macrophages use this pathway to internalize EVs. The process begins with the deformation of the cell membrane surrounding the EVs that have contacted the membrane, subsequently internalizing them into the lysosome.
- Macropinocytosis: This is a less common uptake pathway used by the cell to capture extracellular fluids. It requires actin-driven lamellipodia to initiate invagination into the plasma membrane [2,103,104].

#### 8.1.3. Receptor–Ligand Interaction

**Figure 5:**
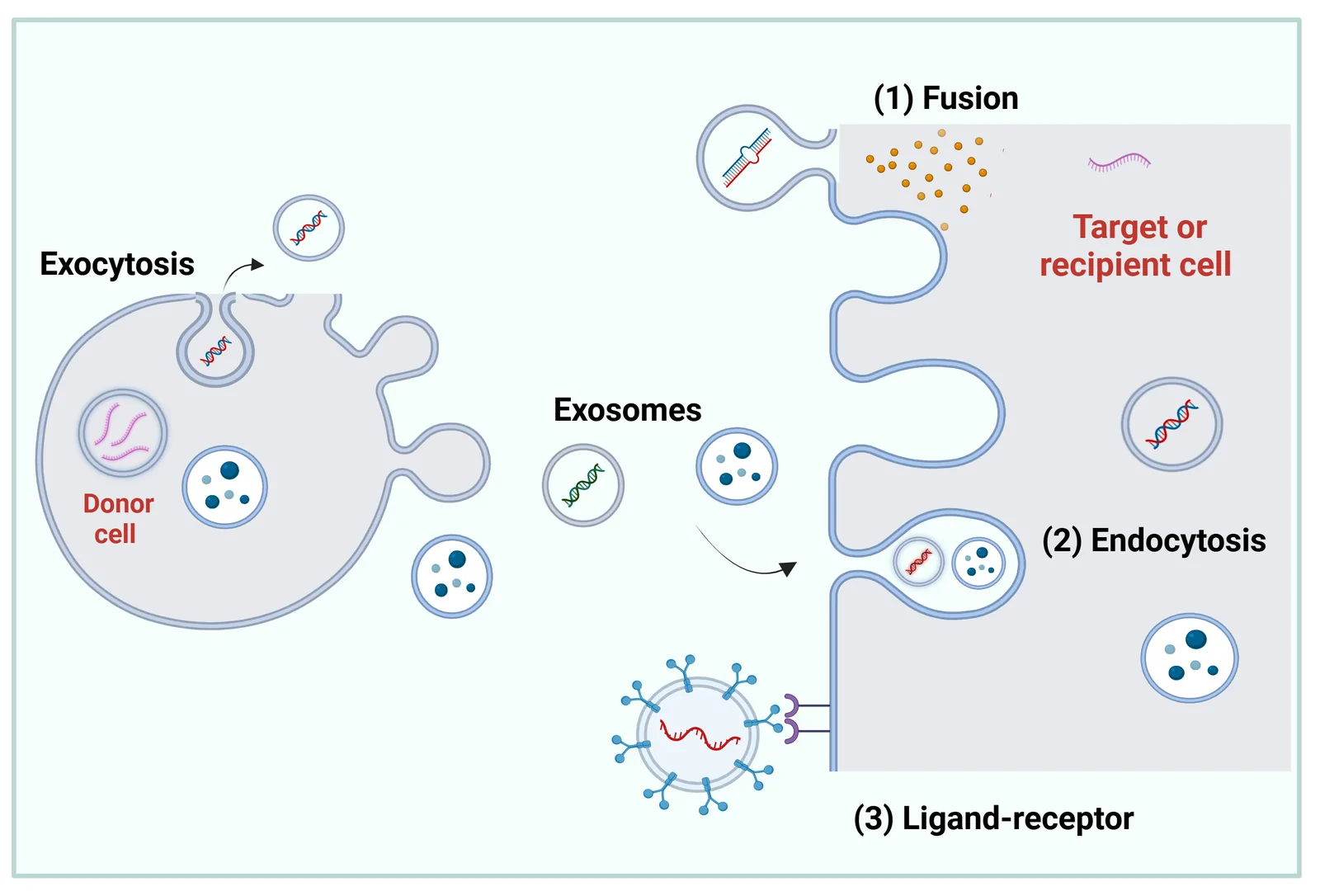
Mechanisms of exosome secretion and uptake. “Created in BioRender. Mateos, F. (2026) https://BioRender.com/6gzp0o9”.

Surface components on the exosome membrane including proteins, lipids, and glycoproteins can bind to specific surface receptors on the recipient cell, directly activating intracellular signaling cascades. Prominent players in these yuxtacrine interactions include tetraspanins, integrins, major histocompatibility complexes (MHC), intercellular adhesion molecule 1 (ICAM-1), proteoglycans, and lectins [2,104]. Accumulating evidence suggests that exosome targeting can be a highly selective process, governed by matching ligand–receptor pairs on both the vesicle and target cell membrane [104]. A classic example is the interaction between milk-derived exosomes and monocyte-derived dendritic cells, which is mediated by the binding of exosomal MUC1 to DC-SIGN molecules expressed on the recipient cell surface. Strikingly, exosomes from other cellular sources lacking MUC1 expression fail to internalize into these dendritic cells [108]. Consistent with this receptor-mediated selectivity, competitive inhibition using RGD (Arg-Gly-Asp) peptides which competitively block cell surface integrins significantly diminishes exosome uptake in dendritic cell [109] (Figure 5).

However, EV internalization should be viewed as a cooperative matrix of pathways rather than a singular event. Functional studies must avoid generalized assumptions and verify the specific, overlapping uptake configurations unique to their chosen therapeutic cell models.

### 8.2. Factors Affecting Exosome Uptake by Target Cells

EV internalization can be modulated by several factors, particularly the specific molecules expressed on the vesicular surface that interact with the target cell membrane. Among these, surface proteins play a critical role; for instance, treating exosomes with proteinase K significantly reduces their uptake by ovarian cancer cells, demonstrating the necessity of protein-mediated interactions [110].

Tetraspanins represent a key class of proteins involved in these vesicle-cell interactions, well-known for their roles in cell adhesion, motility, activation, and proliferation. Specifically, Tetraspanin-8 (Tspan8) has been reported to form a complex with the integrin CD49 on exosomes, which facilitates their internalization by endothelial cells and subsequently promotes cell proliferation and migration [111].

Other proteins are similarly vital for exosome internalization. For example, blocking CD11a or its ligand, Intercellular Adhesion Molecule 1 (ICAM-1), which anti-integrin antibodies markedly reduced sEVs uptake in dendritic cells. Comparable decreases in internalization have been observed when blocking the integrins αv (CD51) and β3 (CD61) on the surface of dendritic cells [38,103]. Additionally, incubating macrophages with an anti-TIM4 antibody a receptor essential for phosphatidylserine-dependent phagocytosis significantly diminishes exosome uptake [38,103].

Beyond surface molecules, pharmacological inhibition of endocytic pathways also alters uptake dynamics. For example, chlorpromazine prevents the formation of clathrin-coated pits in the plasma membrane, thereby inhibiting clathrin-mediated endocytosis and decreasing exosome internalization in recipient ovarian cancer cells [110]. Furthermore, sphingolipids, which are essential components of exosomal membranes, are required for the assembly of lipid rafts that participate in clathrin-independent endocytosis [2]. Consequently, pretreating stem cells with a sphingolipid inhibitor has been shown to disrupt exosome uptake by dendritic cells.

### 8.3. Mechanistic Criteria for Validating True Therapeutic Targeting

To claim true in vivo therapeutic targeting rather than simple non-specific cell uptake, engineered sEVs must satisfy four core pharmacological criteria: (1) Receptor Dependency: Prove that uptake is specific, saturable, and ligand-mediated using competitive inhibition (e.g., neutralizing antibodies) or receptor-knockout models. (2) Modified Systemic Biodistribution: Demonstrate altered pharmacokinetics in vivo by avoiding liver/spleen clearance (mononuclear phagocyte system) and shifting accumulation toward the target disease site. (3) Target-Cell Enrichment: Verify preferential binding to target cells within complex, heterogeneous environments (e.g., organoids or post-in vivo cell sorting) rather than adjacent non-target tissue cells. (4) Functional Cargo Delivery: Confirm that payload escapes endo-lysosomal degradation and reaches the cytosol/nucleus intact to exert its intended therapeutic effect (e.g., gene knockdown or phenotypic switch) [9,112].

### 8.4. Methodological Evaluation of Preclinical Literature and Adherence to MISEV Criteria

A significant source of inconsistency and stalled translation in molecular medicine stems from the varying degrees of methodological rigor among published preclinical studies. To address this issue, Table 4 summarizes the primary omissions in extracellular vesicles characterization observed in the literature, mapped against the established MISEV criteria [9].

## 9. Potential Therapeutic Applications of Exosomes in Several Diseases

**Figure 6:**
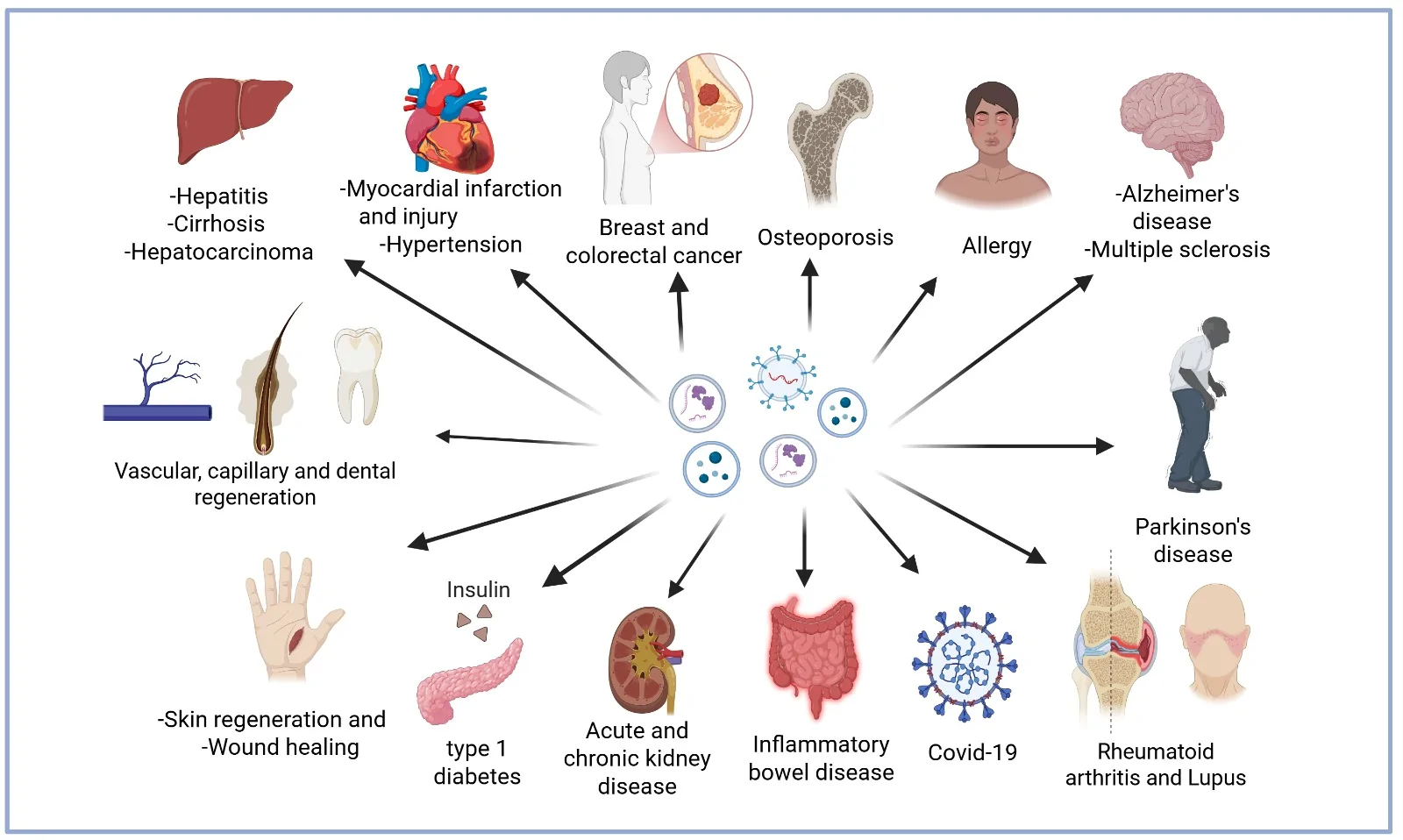
Potential applications of exosomes in the treatment of diseases. “Created in BioRender. Mateos, F. (2026) https://BioRender.com/mr2bhlm”.

sEVs, particularly those derived from mesenchymal stem cells (MSCs), harbor a diverse cargo comprising four major macromolecules classes: nucleic acids, lipids, proteins, and carbohydrates. When administered in various animal models, these biomolecules exert robust beneficial biological effects. These intrinsic therapeutic properties, combined with the high yield and straightforward isolation of exosomes, position them as a highly promising therapeutic platform. To date, sEV-based therapies have been successfully evaluated across a broad spectrum of pathologies, including endocrine, gastrointestinal, cardiovascular, neurodegenerative, pulmonary, renal, and hepatic disorders, as well as in osteoarthritis, wound healing, and cancer. Furthermore, they have demonstrated significant efficacy in managing diabetes mellitus and its secondary complications, such as nephropathy, retinopathy, and diabetic neuropathy, as well as polycystic ovary syndrome [2,5,11,100,113,114,115] (Figure 6).

### 9.1. Cardiovascular Conditions

In vitro and in vivo animal models of heart disease have evaluated the therapeutic potential of miRNA-96 delivered by BM-MSC-Ex. These studies demonstrated a robust protective effect against doxorubicin-induced cardiotoxicity by suppressing oxidative stress, inflammation, and cardiac fibrosis. Mechanistically, this cytoprotection is driven by the inhibition of the NF-κB pathway via the direct targeting and blockade of the *Rac1* gene, which encodes a key upstream activator of this signaling cascade [66].

Other preclinical studies targeting cardiovascular conditions such as myocardial injury, ischemic heart disease, and ischemia/reperfusion (I/R) injury reported that treatment with rat BM-MSC-Ex suppressed myocardial damage by preserving cardiomyocyte viability and function. This protective mechanism was characterized by the downregulation of apoptotic protease activating factor 1 (Apaf-1) and the concomitant upregulation of autophagy-related protein 13 (ATG13). Consequently, this intervention attenuated myocardial apoptosis and fibrosis, thereby mitigating cardiac hypertrophy and preserving cardiac function under pressure overload conditions [116,117]. Furthermore, in a rat model of myocardial infarction induced by ischemic injury, exosome administration promoted neovascularization, enhanced local blood flow, and significantly reduced the localized inflammatory response [118].

Cardioprotective effects have also been recapitulated in murine models of acute I/R injury, where treatment with AMSC-Ex effectively prevented myocardial necrosis and apoptosis [119], Consistent with these findings, similar protective outcomes were observed when isolated cardiomyocytes were exposed to severe oxidative stress in vitro [120].

### 9.2. Bone Conditions

Several studies have reported that the use of BM-MSC-Ex from healthy subjects enhances osteogenesis and suppresses adipogenesis. Conversely, exosomes derived from the BM-MSCs of patients with diabetes harbor high levels of miR-221 and exhibit the opposite effect, leading to a bone–fat imbalance. This highlights exosomal miR-221 as a key regulator and a potential therapeutic target for diabetic osteoporosis [67].

Another preclinical study in diabetic rats demonstrated that treatment with AMSC-Ex enhances bone fracture repair. This therapeutic effect is likely mediated by the activation of the Wnt3a/β-catenin signaling pathway which drives the osteogenic differentiation of BM-MSC [70]. Similarly, when male Sprague-Dawley rats with bone fractures was treated with UC-MSC-Ex, fracture healing was significantly accelerated via the Wnt signaling pathway. This intervention resulted in improved apposition at the fracture site and the formation of a continuous cortical bone structure. At the molecular level, UC-MSC-Ex treatment upregulated the protein expression of both β-catenin and Wnt3a. Furthermore, it elevated the gene expression of runt-related transcription factor 2 (RUNX2) a master regulator of bone formation. Consequently, this drove the expression of downstream bone matrix-forming proteins, including type I collagen and osteopontin, which are critical for bone maturation and mineralization [74].

### 9.3. Liver Conditions

In a rat model of liver fibrosis, the administration of BM-MSC-Ex promoted liver regeneration, improved hepatic function, attenuated fibrosis, and suppressed inflammation. At the molecular level, these exosomes inhibited the expression of components of the Wnt/β-catenin pathway, such as (PPARγ, Wnt3a, Wnt10b, β-catenin, WISP1, Cyclin D1), α-SMA, and Collagen I [121]. Consistent with these findings, UC-MSC-Ex treatment in a carbon tetrachloride-induced liver fibrosis model similarly reduced the expression of critical profibrogenic molecules, including Collagen I, Collagen III, TGF-β1, and phosphorylated Smad2 [72].

### 9.4. Wound Healing and Skin Regeneration

In a cutaneous wound healing model, analyzing the effects of exosomes released from MSCs pretreated with neonatal serum revealed significant proangiogenic activities in endothelial cells, which were driven via the AKT/eNOS signaling pathway [122].

Similarly, in a murine wound healing model, administration of UC-MSC-Ex demonstrated that exosomal miR-21, miR-23a, miR-125b, and miR-145 successfully inhibited the expression of TGF-β2, TGF-βR2, SMAD2, and α-SMA. This coordinated downregulation effectively reduced excessive Collagen I deposition, thereby mitigating scar formation [123].

Furthermore, the capacity of UC-MSC-Ex to enhance angiogenesis has been documented in a rat model of deep skin burns. In this setting, exosome treatment activated the Wnt4/β-Catenin pathway promoting the nuclear translocation of β-Catenin. This nuclear influx subsequently up-regulated the expression of proliferating cell nuclear antigen (PCNA), cyclin D3, N-cadherin and β-catenin, while concurrently down-regulating E-cadherin levels [73].

### 9.5. Kidney Conditions

In a porcine model of metabolic syndrome and renal artery stenosis, the administration of AMSC-derived extracellular vesicles attenuated inflammation by regulating IL-10 expression and concurrently reducing levels of IL-6, IL-1β and TNF-α in the renal vein, thereby improving renal function and mitigating fibrosis [124].

Similarly, in a murine model of chronic kidney disease (CKD), treatment with exosomes derived from melatonin-stimulated AMSCs upregulated the expression of specific microRNAs, including miR-29b-3p, let-7a-3p, let-7b-5p, let-7c-3p, miR-153-3p, miR-26a-2-3p, and miR-846-5p. This exosomal cargo exerted robust anti-inflammatory and antifibrotic effects, characterized by a reduction in serum TNF-α and TGF-β levels, along with a decrease in renal fibrosis driven by the apoptosis of myofibroblasts. Concurrently, a down-regulation of miR-4270, miR-4739, miR-320c, and miR-572 which are typically associated with CKD progression was observed [88].

sEV-based therapies have also demonstrated efficacy in obstructive nephropathies. In a murine model of irreversible unilateral ureteral obstruction (UUO), UC-MSC-Ex administration into the left renal artery mitigated renal fibrosis, significantly lowered serum blood urea nitrogen (BUN) and creatinine levels and suppressed both apoptosis and oxidative stress. Furthermore, an in vitro study using NRK-52E cells stimulated with TGF-β1 showed that co-incubation with UC-MSC-Ex inhibited apoptosis by blocking the ROS-mediated p38MAPK/ERK signaling pathway [75]. Another study evaluating the effects of UC-MSC-Ex on renal fibrosis in a UUO model found that exosome administration prevented renal damage by promoting the ubiquitination and degradation of YAP, a key co-activator associated with TGF-β1 signaling. By targeting YAP for degradation, the downstream transcriptional activity of the Smad2/3 complex was restricted, reducing renal fibrosis [76].

### 9.6. Diabetes Mellitus

In a rat model of type 2 diabetes mellitus (T2DM) induced by a high-fat diet and streptozotocin (STZ), treatment with UC-MSC-Ex successfully restored glucose homeostasis by decreasing blood glucose levels. This therapeutic effect was accompanied by increased glucose uptake in muscle tissue and upregulated expression of glucose transporter 4 (GLUT4). Furthermore, UC-MSC-Ex was shown to enhance insulin sensitivity both in vivo and in vitro via activation of the canonical insulin/AKT signaling pathway. Notably, UC-MSC-Ex administration also promoted insulin secretion and pancreatic islet regeneration by inhibiting STZ-induced cell apoptosis [125].

Correspondingly, in a model of type 1 diabetes mellitus where STZ was utilized to induce islet cell destruction in male Wistar rats, repeated dosing of exosomes derived from menstrual blood-derived mesenchymal stem cells led to a significant increase in β-cell mass and insulin production within the pancreatic islets. This intervention orchestrated islet regeneration via the pancreatic and duodenal homeobox 1 (Pdx-1) pathway, a master transcription factor in β-cell maturation survival, and insulin production [126].

### 9.7. Cancer

The application of sEVs in oncology presents a profound biological paradox. For successful clinical translation, it is imperative to distinguish between the endogenous, disease-perpetuating pro-tumorigenic activities of native EVs and the engineered, therapeutic anti-tumorigenic strategies designed to combat malignancies.

MSC-EVs have been reported to modulate the tumor microenvironment and angiogenesis through dual, context-dependent effects. BM-MSC-Ex promote tumorigenesis and angiogenesis in gastric and colon cancers via the ERK1/2 and p38 MAPK pathways; conversely, in a mammary adenocarcinoma model, BM-MSC-Ex exert a suppressive and anti-angiogenic role through the downregulation of VEGF mediated by specific microRNAs. Other studies on MSC-EVs have shown antiproliferative and pro-apoptotic effects in various tumor models. BM-MSC-EVs induce apoptosis, necrosis, and cell cycle arrest in hepatocellular carcinoma, ovarian cancer, and Kaposi sarcoma. However, pro-proliferative and oncogenic effects have been observed in various tumor types, including nasopharyngeal carcinoma, osteosarcoma, lung, breast, kidney and cancer, following MSC-EVs administration. These processes are mediated by the transfer of specific microRNAs, such as miR-410 and miR-130b-3p, which modulate key molecular pathways, including PTEN inhibition and the FOXO3/NFE2L2/TXNRD1 axis [127].

BM-MSC-Ex and exosomes promote tumor dormancy and chemoresistance in metastatic breast cancer models. This phenomenon is triggered by the transfer of miR-23b and the induction of the EMT [127].

This dual behavior may be associated with MSC heterogeneity, the tumor microenvironment, the cancer type, and differences in experimental conditions. Multiple mechanisms and the specific cargo of the EVs themselves may play a role in modulating tumor progression. Listed below are some of the therapeutic effects observed in various studies of cancer involving sEVs [127].

Exosomes derived from MSC can enhance the sensitivity of malignant cells to chemotherapeutic agents [101]. This chemosensitizing property was demonstrated in an in vivo study of hepatocellular carcinoma (HCC), where the intratumoral administration of exosomes derived from miR-122-modified adipose tissue MSCs significantly increased the therapeutic efficacy of sorafenib against malignant cells [128]. Beyond stem cell sources, macrophage-derived exosomes loaded with paclitaxel successfully inhibited the growth of lung metastases in a murine model. Notably, this formulation markedly increased cytotoxicity in multidrug-resistant MDCK-MDR1 cells overexpressing P-glycoprotein (P-gp), highlighting the potential of exosome-mediated delivery to bypass classic drug resistance mechanisms [129].

Other therapeutic effects of exosomes are mediated by their miRNA cargo, whether natively expressed or artificially incorporated via specific loading methods. For example, a study demonstrated that ADMSC-Ex loaded with miR-199a efficiently inhibited the mTOR signaling pathway in HCC, thereby enhancing tumor sensitivity to doxorubicin [130]. Similarly, in prostate cancer, BM-MSC-Ex carrying miR-205 were shown to delay cancer progression. Mechanistically, this exosomal miRNA targets and inhibits the Rho GTPase-activating protein 2 (RHPN2) gene, thereby suppressing tumor cell proliferation, migration, and invasion while inducing apoptosis. These findings highlight miR-205 as both a promising prognostic biomarker and a potential therapeutic target for prostate cancer [131].

### 9.8. Other Conditions

Rats with brain injury induced by middle cerebral artery occlusion/reperfusion (MCAO/R) were treated with BM-MSC-Ex previously transfected with miR-223-3p. This treatment led to a significant reduction in cerebral infarct volume, marked improvement in neurological deficits, and the suppression of pro-inflammatory cascades mediated by microglial M1 polarization, alongside enhanced secretion of anti-inflammatory factors within the ischemic cortex. This protective effect was linked to the inhibition of signaling mediated by cysteinyl leukotriene receptor 2 (CysLT2R), an upstream receptor strongly associated with ischemic brain injury [132]. Furthermore, parallel studies have highlighted the therapeutic role of miR-125b-5p enriched within AMSC-Ex. This specific microRNA participates in the repair of ischemic muscle tissue by modulating the function of alkaline ceramidase 2 (ACER2). In diabetic ischemia models, ACER2 regulation is crucial, as its dysregulation is directly associated with the overproduction of reactive oxygen species (ROS) and subsequent oxidative damage [71].

## 10. Systemic EV Delivery vs. Direct EV Delivery

A major obstacle in translating preclinical sEVs data into clinical success is the profound pharmacokinetic mismatch between small rodents and humans. Preclinical studies often report remarkable therapeutic efficacy largely due to the administration of disproportionately high localized or systemic doses in rodents, a dosing strategy unfeasible in human clinical trials, where systemic administration predominates. Furthermore, rodent models enable detailed quantitative tracking via fluorescent labeling or radiolabeling, techniques that cannot be routinely applied to human subjects. Following systemic injection, 80% to 90% of the administered sEV dose is rapidly cleared within 5 to 30 min through phagocytosis by resident macrophages (Kupffer cells in the liver and marginal zone macrophages in the spleen). An additional 5% to 8% rapidly accumulates in the lungs and kidneys due to mechanical entrapment and renal clearance pathways. Consequently, less than 1% to 5% of the injected sEVs successfully reach target peripheral tissues, such as ischemic myocardium, injured liver parenchyma, or solid tumors. Achieving a therapeutic window in humans therefore demands excessively high sEVs doses, escalating manufacturing costs, biological safety risks, and off-target toxicity. To circumvent these pharmacokinetic limitations, bioengineering strategies such as decorating sEVs surfaces with anti-phagocytic signals like CD47 (“don’t eat me” markers) are increasingly employed to evade hepatic clearance and lower the required therapeutic threshold [112,133].

## 11. Exosomes as Vehicles for Therapeutic Cargo Delivery

Recent research has highlighted the utility of sEVs as versatile nanocarriers for a wide array of therapeutic moieties, demonstrating outstanding efficacy in delivering proteins, nucleic acids, and small-molecule drugs. To introduce these agents into the vesicles, various loading strategies have been established, among which electroporation, sonication, passive incubation (diffusion), and freeze–thaw cycles are the most widely utilized [14,134] (Table 5).

The primary traits that justify the deployment of sEVs as targeted drug delivery systems include their high stability, low immunogenicity, innate membrane permeability, homing specificity (tissue tropism), targeted delivery capacity, low toxicity and excellent biocompatibility [135,136]. Utilizing them as transport vehicles offers significant clinical advantages, such as enhancing drug stability against enzymatic degradation in biological fluids, improving the bioavailability of poorly soluble compounds through the encapsulation of hydrophobic drugs, and enabling targeted therapy to specific cell populations [3,13,15,137,138]. Furthermore, owing to their nano-scale size and unique physiological properties, sEVs can successfully traverse the blood–brain barrier (BBB), offering a promising avenue for targeted drug delivery to the central nervous system [139].

### Potential Applications of Loaded Exosomes in Medicine

The neuroprotective effects tested in an in vitro model of neuroinflammation, performed in a human microglial clone 3 (HMC3) cell line, where the cells were stimulated with LPS to induce inflammation and previously treated with quercetin-loaded exosomes, showed a reduction in the expression and secretion of pro-inflammatory cytokines, as well as a suppression of nitric oxide production, effects that were attributed to the inhibition of NF-κB [140]. In another pre-clinical models of Parkinson’s disease, exosomes derived from monocytes and macrophages were loaded with catalase, a potent antioxidant enzyme. These engineered vesicles were readily internalized by neuronal cells in vitro and extensively localized within the brains of mice with Parkinson’s disease, eliciting robust neuroprotective effects both in vitro and in vivo [141].

From the perspective of complications arising from Diabetes Mellitus, bovine spleen leukocyte-derived exosomes (IMMUNEPOTENT CRP) loaded with gentamicin, after their application, accelerated wound healing and combat infections in infected diabetic ulcers in a murine model. This treatment improved tissue regeneration; at the molecular level, it enhanced the PI3K-AKT pathway and modulated the inflammatory response, reducing levels of proinflammatory cytokines and increasing levels of IL-10 [142].

A successful study in which a delivery system was developed from quercetin-loaded exosomes, which prevented myopia by mitigating scleral remodeling, matching the effectiveness of 0.1% atropine in reducing axial elongation and refractive error. This treatment improved bioavailability and precorneal retention time of the quercetin, enhancing its efficacy, relieving endoplasmic reticulum stress, and suppressing ferroptosis with high biosafety [143].

An icariin-loaded adipose stem cell-derived exosomes system (AMSC-Ex-Ica) was used to treat collagen-induced rheumatoid arthritis in a murine model. AMSC-Ex-Ica cells accumulated in joints, reducing synovitis and preserving cartilage. This treatment promoted the transition of inflammatory (M1) to anti-inflammatory (M2) macrophages in vitro and in vivo, and reduced glycolysis in macrophages by inhibiting the ERK/HIF-1α/GLUT1 metabolic pathway, thereby attenuating inflammation [144].

Curcumin is a natural polyphenol with well-documented therapeutic benefits in inflammatory diseases; however, its clinical potential is severely hindered by low aqueous solubility and poor in vivo stability. To overcome these limitations, albumin-bound curcumin was encapsulated into extracellular vesicles (CA-VE) by gentle sonication for use on cutaneous inflammation in vitro and in vivo. This formulation significantly enhanced the stability of curcumin, facilitating efficient intracellular internalization without inducing cytotoxic effects, and robustly suppressed inflammation both in vitro and in vivo [145].

Estrogen replacement remains one of the primary therapeutic strategies for osteoporosis. Consequently, a study evaluated BM-MSC-Ex loaded with 17β-estradiol in BM-MSC cultures. A significantly higher cell survival rate was observed in the treated group compared to the control, suggesting that estradiol-loaded exosomes hold strong potential as biocompatible nanocarriers for osteoporosis therapeutics [146].

Furthermore, macrophage-derived exosomes loaded with paclitaxel effectively inhibited the growth of lung metastases in a murine model. This nano-formulation markedly increased cytotoxicity in multidrug-resistant MDCK-MDR1 cells overexpressing P-glycoprotein (P-gp), exerting a potent anticancer effect [129].

Similarly, in a murine model of hepatic fibrosis, treatment with BM-MSC-Ex loaded with luteolin, a flavonoid with potent antioxidant and anti-inflammatory properties demonstrated superior antifibrotic activity compared to either free luteolin or naïve exosomes alone, highlighting this approach as a promising intervention for liver fibrosis [138].

Finally, targeted gene therapy approaches have also leveraged exosomal delivery; an antisense miRNA oligonucleotide against miR-21 (AMO-21) was encapsulated into modified exosomes displaying the T7 peptide (T7-exo). In vitro, T7-exo demonstrated significantly higher uptake efficiency into C6 glioblastoma cells compared to unmodified exosomes. In vivo, the administration of T7-exo loaded with AMO-21 to rats with intracranial glioblastoma resulted in superior tumor targeting, efficient downregulation of oncogenic miR-21 levels, and a substantial reduction in tumor volume [147] (Table 5).

**Table 5:** Applications of Loaded Exosomes in the Treatment of Several Diseases.

Origen	Loaded Molecule	Loading Method	Loading Method Conditions	Therapeutic Effects/Advantages	Reference
UC-MSC	Quercetin	Sonication	20% amplitude, 6 cycles (3 s on/3 s off) for 3 min, 2 min cooling/cycle. Incubation at 37 °C for 1 h.	Delivering quercetin in a stable and bioavailable form via exosomes enhances its cellular uptake and anti-inflammatory potency.	[140]
Exosomes derived from a bovine leukocyte spleen	Gentamicin	Electroporation	Electroporation at 250 V and 125 μF followed by incubation at 37 °C for 30 min.	Enhances the therapeutic efficacy of gentamicin in *S. aureus*-infected diabetic wounds.	[142]
UC-MSC	Quercetin	Incubation	37 °C for 2 h.	The system improves quercetin solubility, corneal permeability, and precorneal retention time, thereby enhancing therapeutic efficacy.	[143]
AMSC	Icariin	Incubation	Room temperature for 2 h.	Significantly enhances the anti-rheumatoid arthritis efficacy of AMSC-Ex-Ica.	[144]
J774A.1	Albumin and curcumin	Sonication	20% amplitude, 6 cycles (3 s on/3 s off) for 3 min, 2 min cooling/cycle.	Improves curcumin stability, shows efficiency in vitro cellular internalization of CA-EVs with minimal cytotoxicity, and decreases inflammation.	[145]
BM-MSC	17β-estradiol	Incubation	37 °C for 1 h in shaker.	Enhances drug stability and cell survival.	[146]
		Sonication	20% amplitude, 6 cycles (3 s on/3 s off) for 3 min, 2 min cooling/cycle. Incubation at 37 °C for 1 h.		
Macrophages	Paclitaxel	Incubation	37 °C for 1 h in shaker.	Exosomes co-localization and potent anticancer efficacy.	[129]
		Electroporation	1000 kV for 5 ms, followed by incubation at 37 °C for 30 min.		
		Sonication	20% amplitude, 6 cycles (3 s on/3 s off) for 3 min, 2 min cooling/cycle. Incubation at 37 °C for 1 h.		
BM-MSC	Luteolin	Incubation	37 °C × 1 h in shaker.	Sustained drug release in circulation, exerting antioxidants, anti-inflammatory, and immunomodulatory effects against hepatic fibrosis.	[138]
		Sonication	20% amplitude, 10 cycles (3 s on/3 s off) for 3 min, 2 min cooling/cycle. Incubation at 37 °C for 1 h.		
Macrophages	Catalase	Incubation	Room Temperature for 18 h.	Sustained release and protease stability of catalase. Efficient neuronal uptake in vitro and in vivo brain targeting in Parkinson’s disease mice, demonstrating neuroprotective effects.	[141]
		Sonication	500 V, 2 kHz, 20% power, 6 cycles (4 s pulses/2 s pauses).		
		Freezing/thawing	3 cycles (Freezing/thawing −80 °C/RT).		
		Extrusion	Pore size 200 nm.		
293T	Inhibitor of miR-21	Electroporation	400 V	More efficient cellular uptake of AMO-21 than unmodified exosomes, significantly reducing miR-21 levels and tumor volume in glioblastoma.	[147]

## 12. Exosome-Based Therapies in Clinical Trials

The therapeutic potential of sEVs in tissue regeneration, coupled with innovative bioengineering approaches that enhance their homing and drug-loading capabilities, has driven a significant increase in the number of clinical trials evaluating these vesicles. To map the current clinical landscape, a comprehensive search was conducted on ClinicalTrials.gov "https://clinicaltrials.gov" (accessed on 20 July 2026), filtering for interventional studies that utilized sEVs as a primary therapeutic agent across diverse human pathologies.

Oncological applications represent a major focus area, with active registered trials targeting breast, prostate, and colorectal cancers. Furthermore, leveraging the innate capacity of sEVs to traverse the BBB, several studies target central nervous system disorders, including neurodegenerative pathologies such as Alzheimer’s disease, Parkinson’s disease, and multiple sclerosis. In the cardiovascular domain, clinical investigations primarily address acute ischemic stroke, myocardial infarction and hypertension. Additionally, the scope of exosomal interventions spans dermatological conditions (e.g., androgenetic alopecia and diabetic foot ulcers), metabolic disorders (e.g., obesity and types 1 and 2 diabetes mellitus), as well as respiratory and infectious diseases, among which COVID-19 was one of the most frequent conditions. Notably, the majority of these included trials are restricted to interventional designs and reside in early clinical development, predominantly in Phase 1 and Phase 2 testing (Table 6), ref. [148].

## 13. Challenge in Small EV-Based Therapeutics

Despite the immense therapeutic potential of sEVs as cell-free biotherapeutics and drug delivery vectors, several critical technicals, biological, and regulatory bottlenecks must be resolved before wide-scale clinical implementation can be realized.

### 13.1. Structural and Cargo Heterogeneity

One of the most prominent obstacles is the inherent heterogeneity of EVs preparations. sEVs exhibit massive diversity at three distinct levels: cell-source heterogeneity (variations between donor cells), batch-to-batch heterogeneity (variations between manufacturing runs), and intra-sample heterogeneity (co-existence of distinct vesicle subpopulations with different sizes, membrane lipids, and protein markers within a single isolate). This multi-layered diversity directly impairs pharmacological reproducibility, making it exceedingly difficult to guarantee consistent therapeutic payloads and identical biological potencies between different treatment batches [149,150,151].

### 13.2. Scalability and Industrial Manufacturing

Transitioning sEV production from small-scale laboratory settings to large-scale, industrial-grade Good Manufacturing Practice (GMP) standards presents a major bioengineering challenge. Conventional benchtop isolation techniques, such as differential ultracentrifugation, are entirely unsuited for industrial scale-up due to their low throughput and operational complexity. While advanced technologies like Tangential Flow Filtration (TFF) offer scalable options, optimizing upstream cell culture conditions (e.g., utilizing large-scale bioreactors and serum-free media) without altering the natural therapeutic properties of the cells or triggering cellular stress remains technically demanding and costly [149,150,151,152].

### 13.3. Pharmacokinetics and Biodistribution

The physiological behavior of intravenously administered sEVs presents a significant pharmacological barrier. The biodistribution of unmodified vesicles revealed rapid clearance and preferential accumulation in organs of the mononuclear phagocyte system (liver, lungs, and spleen)—minimizing accumulation in target tissues—followed by rapid elimination and/or phagocytosis that prevents the functional release of the therapeutic cargo. Modifying the vesicle surface with hydrophilic groups, such as PEG, via bioengineering techniques prolongs the stability and circulating half-life of these sEVs [149,150,151].

### 13.4. Safety and Immunogenicity of EVs

The safety of sEV-based therapies in early-stage clinical trials is a matter of significant importance. MSC-derived exosomes exhibit low immunogenicity compared to cell-based therapies; while they possess a favorable biological safety profile due to their biocompatibility, the presence of cellular debris or microbial contaminants such as endotoxins could trigger unwanted immune responses, meaning that their potential adverse effects and long-term consequences still require exhaustive investigation. Coupled with this, current research focuses on acute pathology models; therefore, further studies in animal models are needed to evaluate treatments for chronic pathological conditions in order to assess cumulative effects, off-target effects, and potential long-term toxicity [149,150].

### 13.5. Clinical Translation and Regulatory Frameworks

Agencies such as the U.S. Food and Drug Administration (FDA) and the European Medicines Agency (EMA) classify EVs-derived therapies as biological products; consequently, from a regulatory standpoint, sEVs used in clinical research studies must be manufactured in compliance with GMP standards throughout the various stages of production. Therefore, standardized, reproducible, and scalable manufacturing processes are required for the successful clinical translation of these therapies. Establishing GMP-compliant laboratories thus entails significant financial investment and infrastructure development.

The absence of universally accepted criteria for defining absolute dose metrics (e.g., particle vs. protein vs. potency counts), standardized validation of batch purity, and validated high-throughput quality control assays creates an uncertain environment that delays the initiation and approval of human clinical trials [149,152].

### 13.6. Current Dosing Paradigms in sEV Therapeutics

The clinical translation of sEV therapeutics is hindered by the lack of standardized dosing nomenclature, compromising cross-study comparability and regulatory approval. Current quantification approaches rely on distinct analytical paradigms, each presenting technical limitations:

- Protein-Based Dosing: Measures total protein via colorimetric assays; highly accessible but prone to overestimating doses due to co-isolated non-vesicular contaminants (e.g., albumin).
- Particle-Based Dosing: Utilize Nanoparticle Tracking Analysis (NTA) and Resistive Pulse Sensing (RPS); provides entity counts but fails to distinguish intact sEVs from non-vesicular aggregates or lipid droplets of similar size.
- Source-Cell Normalization: Correlates dose with producing-cell counts; useful for bioprocess scaling but ignores batch-to-batch variations in secretion rates.
- Potency-Based Dosing: Quantifies dosage via functional bioassays aligning with FDA/EMA standards; highly labor-intensive yet offers superior therapeutic reproducibility [9,153].

Translational Perspective to ensure clinical reproducibility and satisfy regulatory requirements, emerging consensus advocates for a multi-parametric framework integrating total protein concentration, absolute particle count, and validated functional potency units.

## 14. Conclusions

sEVs represent a revolutionary and cutting-edge platform within the fields of regenerative medicine and cell-free therapies. As natural vectors of intercellular communication, these nanovesicles possess the intrinsic capability to transfer a complex and dynamic biological cargo comprising proteins, lipids, and various nucleic acids that can precisely modulate critical signaling pathways and selectively reprogram the function of recipient cells. The robust preclinical evidence compiled throughout this review demonstrates their multifaceted efficacy in treating cardiovascular, bone, hepatic, renal, metabolic, and oncological pathologies, where they actively promote tissue repair and regeneration and immunomodulation.

Furthermore, key properties such as high stability, low immunogenicity, excellent biocompatibility and low toxicity coupled with the feasibility of manipulating their content through microenvironmental preconditioning or advanced cargo bioengineering, establish them as a highly efficient and targeted drug delivery system. Notably, their unique ability to cross complex biological barriers, including the blood–brain barrier, opens new frontiers for the treatment of central nervous system disorders. Although the inherent heterogeneity in their isolation and purification methods still poses considerable technical and regulatory challenges that require standardization, a growing number of early-phase clinical trials are evaluating their potential clinical viability. Ultimately, exosomes have transitioned from being regarded as mere cellular waste intermediates to becoming one of the most promising biotherapeutic and precision delivery systems in modern medicine.

## Figures and Tables

**Table 1:** Characteristics of the different types of extracellular vesicles.

Characteristics	Small EVs (Exosome/Small Ectosome)	Large EVs (Microvesicles/ Ectosome)	Apoptotic Bodies
Typical size range (nm)	30–150	100–1000	500–2000
Condition of parental cell	Physiological and pathological conditions.	Physiological conditions and response to stimulus.	Apoptosis.
Main biogenesis pathway	Endosomal pathway.	Cellular membrane.	Cellular membrane.
Release pattern	Fusion of microvesicles with the cellular membrane.	Outward budding/blebbing of the plasma membrane.	Cell disassembly during apoptosis.
Content	Proteins, mRNA, miRNA and other non-coding RNAs.	Proteins, mRNA, miRNA and other non-coding RNAs.	Nuclear Fraction, cellular molecules.
Cell markers	Alix, Tsg101, tetraspanins (CD81, CD63, CD9), flotillin.	Integrins, selectins, CD40 y metalloproteinases.	Annexin V, phosphatidylserine, histones.
Function	Cellular Communication.	Cellular Communication.	Facilitate phagocytosis.
Limitations	Size and markers overlap significantly with sEVs. Physical dimensions alone cannot definitively confirm an endosomal origin.	No exclusive marker exists. Overlaps in size with both upper-range sEVs and smaller apoptotic bodies.	Highly heterogeneous. Smaller apoptotic bodies can share identical sizes, morphologies with Large EVs

**Table 3:** Isolation methods.

Isolation Methods	Ultracentrifugation	Size-Exclusion Chromatography (SEC)	Ultrafiltration	Precipitation PEG	Immunoaffinity
Principle	Separation based on size and density via sequential centrifugal forces.	Second most widely used method; Based on particle size.	Liquid flows parallel to a filter membrane, separating by size cut-off.	Utilizes a hydrophilic polymer reducing EVs solubility and enabling precipitation via low-speed centrifugation.	Antigen–antibody interactions to isolate components from a mixture. Antibodies against vesicle markers (CD81, CD63, or CD9).
Purity	Intermediate	High	High	Low	High
Time	High	Low	High	Intermediate	High
Cost	Low	Low	Intermediate	Low	High
Yield	Low	High	Intermediate	High	Intermediate
Functionality	Intermediate	High	Intermediate	Intermediate	Low
Advantages	Suitable for processing large initial sample volumes.	Preserves VEs structure and biological activity.	High-throughput capability; rapid filtration cycles.	Simple, rapid, cost-efficient.	High specificity, sensitivity, purity, and yield.
Limitations	High risk of protein aggregate co-precipitation.	Dilutes the final sample, requiring a subsequent concentration step.	Membrane clogging, yield loss.	Lower purity	Expensive, low yield, labor-intensive.
References	[3,6,95,100,102]	[3,6,13,95,100,102]	[4,6,13,95,102]	[4,13,95,100,102]	[3,6,95,100,102]

**Table 4:** Critical Mapping of Preclinical EV Literature Against MISEV Standards.

MISEV Core Requirement.	Technical Objective.	Common Non-Compliance Pattern in Cited Studies.	Impact on Preclinical Reliability and Interpretation.
Category 1: Characterization of transmembrane/GPI-anchored proteins	Presence of lipid-bilayer vesicles (CD9, CD63, CD81).	Single marker reliance without demonstrating surface multiplex panel.	High risk of false positives; overlaps EV subpopulations or subtypes.
Category 2: Characterization of cytosolic/intracellular proteins	Membrane integrity and vesicular nature (Alix, TSG101, Flotillin).	Cytosolic marker omission, relying exclusively on surface antibody stains or physical sizing.	Fails to verify if EVs are intact or simply membrane debris.
Category 3: Demonstration of negative/exclusion control markers	Contamination from intracellular compartments (Calnexin, GM130, Histones).	Complete absence of negative control blotted matrices.	Artifacts may drive bioactive instead of EVs.
Category 4: Assessment of non-vesicular macromolecular purity	Co-isolated soluble components (Albumin in blood, ApoA1).	Evaluating total protein as indicator of EV dose without purity check	Overestimates therapeutic potency; clouds pharmacokinetic evaluation.
Category 5: Dual biophysical characterization	Pair biochemical markers with size and count (NTA + TEM/SEM).	Use only light-scattering metrics (NTA/DLS) without electron microscopy.	Inadequate quantification; NTA does not distinguish between functional sEVs, protein aggregates, and macromolecular droplets.

**Table 6:** Interventional clinical trials utilizing exosomes registered in ClinicalTrials.gov [148].

Nº	NCT Number	Study Title	Study Status	Conditions	Interventions	Phase
1	NCT07372001	Effects on Facial Skin Aging After Topical Application of Exosomes with a Microneedling Device.	Completed	Skin Aging.	Lyophilized exosomes; Topical.	4
2	NCT07105371	Patients With ALS and Other Motor Disorders Will be Treated with Mesenchymal Cell Exosome Solution.	Completed	Motor Disorders.	AlloEx exosomes derived from MSCs; intranasal.	1
3	NCT04276987	A Pilot Clinical Study on Inhalation of Mesenchymal Stem Cells Exosomes Treating Severe Novel Coronavirus Pneumonia.	Completed	Severe Novel Coronavirus Pneumonia.	Exosomes derived from allogenic adipose MSCs; aerosol inhalation.	1
4	NCT06466850	Mesenchymal Stem Cells Derived Exosomes in Osteoarthritis Patients.	Recruiting	Osteoarthritis, Knee.	Exosome derived from MSCs, intra-articular injection.	NA
5	NCT07620158	Proof of Concept Study to Isolate Cosmetic Improvement of Skin Using the Vitro Biopharma Secretome/Exosome Serum.	Completed	Cosmetic Effect.	Secretome/Exosome Serum, extracellular vesicles derived from umbilical cord MSCs.	NA
6	NCT04849429	Intra-discal Injection of Platelet-rich Plasma. Enriched with Exosomes in Chronic Low Back Pain.	Completed	Chronic Low Back Pain/Degenerative Disk Disease.	Platelet rich plasma with exosomes; injection.	1
7	NCT06812637	Efficacy and Safety of Wharton’s Jelly-Derived Mesenchymal Stem Cell Exosomes in the Treatment of Diabetic Foot Ulcers: A Double-blinded Randomized Controlled Clinical Trial.	Completed	Diabetic Foot Ulcer.	Warton jelly derived MSCs derived exosomes.	1
8	NCT06239207	Efficacy and Safety of Exosomes Versus Platelet Rich Plasma in Patients of Androgenetic Alopecia.	Competed	Androgenic Alopecia.	Exosomes; GFC CELL EXO SCALP KIT (Leuco Exo 97%)/Platelet Rich Plasma.	2
9	NCT04602104	A Clinical Study of Mesenchymal Stem Cell Exosomes Nebulizer for the Treatment of Acute Respiratory Distress Syndrome.	Completed	Acute Respiratory Distress Syndrome.	Phase 1: Allogeneic MSC-Ex; Phase 2: Dose 1 and 2 of allogeneic MSC-Ex; aerosol inhalation	1/2
10	NCT02138331	Effect of Microvesicles and Exosomes Therapy on β-cell Mass in Type I Diabetes Mellitus (T1DM).	-	Diabetes Mellitus Type 1.	MSC exosomes.	2/3
11	NCT04388982	The Safety and the Efficacy Evaluation of Allogenic Adipose MSC-Exos in Patients with Alzheimer’s Disease.	-	Alzheimer Disease.	Low, medium, and high doses of AMSC-Ex; intranasal instillation.	1/2
12	NCT01294072	Study Investigating the Ability of Plant Exosomes to Deliver Curcumin to Normal and Colon Cancer Tissue.	-	Colon Cancer.	Curcumin-conjugated plant-derived exosomes.	-
13	NCT01159288	Trial of a Vaccination with Tumor Antigen-loaded Dendritic Cell-derived Exosomes.	Completed	Non-Small Cell Lung Cancer.	Tumor antigen-loaded dendritic cell-derived exosomes; intradermal injection	2
14	NCT03608631	Exosomes in Treating Participants with Metastatic Pancreas Cancer with KrasG12D Mutation.	Recruiting	Metastatic pancreatic cancer.	MSC-Exosome loaded with siRNA against KrasG12D.	1/2
15	NCT05669144	Co-transplantation of Mesenchymal Stem Cell Derived Exosomes and Autologous Mitochondria for Patients Candidate for CABG Surgery.	-	Myocardial Infarction/Myocardial Ischemia.	MSC-Ex combined with autologous mitochondria.	1/2
16	NCT03384433	Allogenic Mesenchymal Stem Cell Derived Exosome in Patients with Acute Ischemic Stroke.	-	Cerebrovascular Disorders.	miR-124-transfected allogeneic MSC-Ex	1/2

## Data Availability

No new data were created or analyzed in this study. Data sharing is not applicable to this article.

## Abbreviations

The following abbreviations are used in this manuscript:

AMSC-Ex	Exosomes derived from mesenchymal stem cells of adipose tissue
AMSCs	Adipose tissue-derived mesenchymal stem cells
BBB	Blood–brain barrier
BM-MSC-EVs	Extracellular vesicles derived from bone marrow mesenchymal stem cells
BM-MSC-Ex	Exosomes derived from bone marrow mesenchymal stem cells
BM-MSCs	Bone marrow-derived mesenchymal stem cells
CME	Clathrin-Mediated Endocytosis
EMA	European Medicines Agency
ESCRT	Endosomal sorting complex required for transport
EVs	Extracellular vesicles
FAS	Signal of apoptosis ligand
FDA	Food and Drug Administration
GMP	Good Manufacturing Practice
HCC	hepatocellular carcinoma
ILV	Intraluminal vesicles
ISEV	International Society for Extracellular Vesicles
LEVs	Large extracellular vesicles
MISEV	Minimal Information for Studies of Extracellular Vesicles
MSCs	Mesenchymal Stem Cells
MSC-sEVs	Extracellular vesicles derived from mesenchymal stem cells
MVB	Multivesicular body
MVs	Microvesicles
RBPS	RNA-binding proteins
sEVs	Small extracellular vesicles
STZ	Streptozotocin
TEM	Transmission electron microscopy
TGF-β1	Transforming growth factor β1
TNF-α	Tumor necrosis factor α
UC-MSC-Ex	Exosomes derived from umbilical cord mesenchymal stem cells
UC-MSCs	Umbilical cord mesenchymal stem cells
Wnt	Wingless proteins
